# Role of Coenzyme Q_10_ in Health and Disease: An Update on the Last 10 Years (2010–2020)

**DOI:** 10.3390/antiox10081325

**Published:** 2021-08-23

**Authors:** Ilenia Cirilli, Elisabetta Damiani, Phiwayinkosi Vusi Dludla, Iain Hargreaves, Fabio Marcheggiani, Lauren Elizabeth Millichap, Patrick Orlando, Sonia Silvestri, Luca Tiano

**Affiliations:** 1 School of Pharmacy, University of Camerino, 62032 Camerino, Italy; ilenia.cirilli@unicam.it; 2 Department of Life and Environmental Sciences, Polytechnic University of Marche, 60131 Ancona, Italy; e.damiani@univpm.it (E.D.); f.marcheggiani@univpm.it (F.M.); l.millichap@pm.univpm.it (L.E.M.); p.orlando@univpm.it (P.O.); s.silvestri@univpm.it (S.S.); 3 Biomedical Research and Innovation Platform, South African Medical Research Council, Tygerberg 7505, South Africa; Phiwayinkosi.Dludla@mrc.ac.za; 4 School of Pharmacy and Biomolecular Sciences, Liverpool John Moores University, Liverpool L3 3AF, UK; i.hargreaves@ucl.ac.uk

**Keywords:** Coenzyme Q, ubiquinol, cardiovascular disease, reproductive medicine, skeletal muscle health, skin health, neurodegeneration, ophthalmology

## Abstract

The present review focuses on preclinical and clinical studies conducted in the last decade that contribute to increasing knowledge on Coenzyme Q_10_’s role in health and disease. Classical antioxidant and bioenergetic functions of the coenzyme have been taken into consideration, as well as novel mechanisms of action involving the redox-regulated activation of molecular pathways associated with anti-inflammatory activities. Cardiovascular research and fertility remain major fields of application of Coenzyme Q_10_, although novel applications, in particular in relation to topical application, are gaining considerable interest. In this respect, bioavailability represents a major challenge and the innovation in formulation aspects is gaining critical importance.

## 1. Introduction

Coenzyme Q_10_ (CoQ), is an endogenous lipophilic quinone, ubiquitous in biological membranes, where it acts as a cofactor of mitochondrial respiratory complexes supporting cellular bioenergetics. Its reduced form (ubiquinol) is endowed with antioxidant activities both as a radical scavenger and also supporting, synergistically, the larger cellular antioxidant network. These two functions, bioenergetic and antioxidant, characterized Coenzyme Q research in the last half of the 21st century, while in the first decades of the new millennium, research has unleashed novel functions of CoQ, highlighting its role in modulating gene expression, mitochondrial function and signaling, with important implications in the senescence process and cell death. These biochemical aspects have been investigated in clinical studies, highlighting the critical role of CoQ in health and disease. The present review focuses on novel research and the clinical findings of the past 10 years and represent an update on our previous reports, published between 2007 [1] and 2010 [2], that have already laid an important foundation in terms of discussing literature related to the clinical aspects and biological properties of CoQ in ameliorating different disease conditions.

PubMed, EMBASE, and Google Scholar databases/search engines were used to identify eligible studies. Included studies had to report on the health effect of CoQ as well as being listed among keywords ubiquinone, ubiquinol, CoQ, and ubidecarenone, and should have been published between 2010 and 2021. A total of 450 articles met the inclusion criteria, and among these, 333 referred to original research articles and 117 were reviews. The most prominent topic was cardiovascular health (21%), fertility (12%) and skeletal muscle health (7%), the latter comprising clinical aspects and physical exercise aspects within physiological conditions and sport nutrition. Bioavailability studies comparing formulations have attracted significant attention (8%), probably driven by a major uprise in the number of ubiquinol formulas claiming enhanced bioavailability as well as other formulas. Altogether, these studies highlight a major limitation of CoQ application in human studies associated with its low bioavailability, in particular to specific tissues, such as the skeletal and cardiac muscle and, consequently, the efforts in improving delivery strategies. Partially associated with this topic, novel aspects receiving increasing interest in the literature, refer to the topical application of CoQ to skin (2%) and ophthalmology (2%).

## 2. Cardiovascular Health

Cardiovascular diseases (CVDs) remain the leading cause of death worldwide, accounting for approximately 32% of all global deaths that occurred in 2019 [3]. Aging represents the main risk factor being consistently linked with pathological modifications such as systemic low-grade inflammation, increased arterial stiffness and endothelial dysfunction, processes that may lead to elevated blood pressure (BP) as well as reduced or diminished left ventricular (LV) function [4]. The latter remains the major characteristic feature of diverse cardiovascular pathologies, such as cardiomyopathies, that may lead to heart failure [5]. Moreover, modifiable risk factors involving lifestyle choices, such as tobacco smoking, harmful use of alcohol, and obesity, which may be induced by physical inactivity and overnutrition, are acknowledged to also play an important role in the pathogenesis of several CVDs. In particular, in the last decade, several reviews, both with a comprehensive or systematic approach, have been published, related to CoQ and its impact on CVD-related complications. For instance, in 2012, Krim and colleagues [6] indicated the gap in evidence that could confirm the benefits of micronutrients, including CoQ, in patients with chronic heart failure. However, a pooled analysis of published literature in 2012, by Gao and colleagues [7] showed that CoQ supplementation could improve endothelial function assessed peripherally by flow-mediated dilation in patients with and without established CVDs. Similarly, a meta-analysis conducted by Fotino and co-workers [8], published in the following year (2013), revealed that CoQ supplementation may improve the ejection fraction in patients with congestive heart failure. In 2014, Kosmas and colleagues [9] affirmed that additional clinical trials are required to definitely determine the effect of statins on chronic congestive heart failure, including how the endogenous levels of CoQ are impacted. In contrast, different review papers published between 2015–2016 [10,11,12,13,14] validated claims that CoQ supplementation can significantly reduce morbidity and mortality in patients with heart failure or cardiovascular events; however, these articles also cautioned on the limitation of clinical studies informing on these positive outcomes. Recent reviewed evidence from our group [15,16] and others, including Suárez-Rivero and co-workers [17], as well as Dragan and colleagues [18], have explored the literature on the diverse therapeutic mechanisms that could be attributed to the beneficial effects of CoQ against CVD-related complications. These mainly involve its strong antioxidant and anti-inflammatory properties, especially the reduced form of CoQ (ubiquinol), which also appears to be less studied in comparison to its oxidized counterpart. These limitations highlight the advantages of the current review, where not just information on the impact of CoQ on CVD-related outcomes is updated, but also implications of dose are discussed. More details on the mechanism of action of CoQ have been provided by preclinical studies using different preclinical experiments using cultured cells or animal models. In HUVECs or H9c2 exposed to elevated concentrations of palmitate or high glucose, to mimic the detrimental effects that metabolic stress has on cardiac physiology, CoQ deficiency has been consistent with the development of oxidative stress [19,20,21]. Precisely, these studies show that CoQ deficiency in such conditions promotes increased ROS generation, and depletion of intracellular antioxidant defense systems, such as uncoupling protein 2 (UCP2), and superoxide dismutase 2 (SOD2), leading to cellular damage. Apparently, in HUVECs subjected to conditions of oxidative stress and inflammation, using stressors such as oxidized low-density lipoprotein (oxLDL) or lipopolysaccharide (LPS), CoQ treatment proves to be effective in suppressing endothelial oxidative injuries [22,23,24,25]. Here, prime mechanisms of protection against oxidative stress include blocking the activities of protein kinase C (PKC)/NADPH oxidase, as well as improving mitochondrial function. In relation to inflammation, Olivieri and colleagues [23] demonstrated that the reduced form of CoQ (ubiquinol) could attenuate LPS-induced microRNA (miR)-146a and interleukin-1 receptor (IL-1R) associated kinase (IRAK-1) modulation but failed to curb interleukin (IL)-6 release. This result is of interest, since emerging clinical data already suggest that CoQ intake can significantly affect the expression of more than 100 different microRNAs related to the development of CVDs [26]. On the other hand, using in vivo models, low plasma levels of CoQ in rats with cardiovascular diseases (congenital and acquired) [27,28] with spontaneous myxomatous mitral valve degeneration (MMVD) has been associated with increased levels of lipid peroxidation and the enhanced severity of congestive heart failure. Indeed, Gvozdjáková and colleagues [29] demonstrated that raised levels of CoQ in plasma and myocardial tissue were linked with reduced malondialdehyde (MDA) (a marker of lipid peroxidation) and improved activity of the mitochondrial electron transport chain. These data suggest that strategies to enhance plasma or cardiac tissue levels of CoQ could prove essential to ameliorate CVD-related complications. Furthermore, several studies have reported on the impact of CoQ in reverting ischemia-reperfusion injury or hypertension in animal models. Presented evidence showed that CoQ administration could block lipid peroxidation products and expressions of inducible/endothelial nitric oxide synthase, and apoptosis [30,31,32,33]. From the current results, it could be hypothesized that therapeutic mechanisms of action for CoQ involves improving endothelial function, in part by enhancing nitric oxide bioavailability in addition to blocking oxidative stress and inflammation. This was, in fact, verified by Kozaeva and colleagues [34], showing that CoQ supplementation improved nitric oxide-mediated vasodilation in Wistar rat aortas. Moreover, Kulyak and co-workers showed that CoQ treatment could protect against the development of myocardial hypertrophy in an experimental model of coronary artery occlusion [35]. Overall, the results from other studies demonstrate that CoQ supplementation could effectively attenuate oxidative stress and inflammation to reduce cardiac remodeling or hinder doxorubicin-induced cardiotoxicity [36,37]. This is in line with studies showing that CoQ supplementation in rats could lower the levels of triglycerides and total cholesterol in apoprotein E deficient mice [38,39]. These findings suggest that, besides directly inhibiting lipid peroxidation products, CoQ can lower plasma cholesterol, thus indirectly preventing its reaction with radicals, and in the process block the development of atherosclerosis and related complications. It is noteworthy that CoQ also appears to play a major role in improving cardiac mitochondrial function in experimental models of CVD [40,41]. This aspect is essential, since mitochondria are known to play an important role in energy generation and calcium homeostasis [42], which are vital processes for an efficient cardiac physiology. Finally, Table 1 summarizes clinical evidence on the impact of CoQ supplementation on human cardiovascular status both in healthy and in CVD subjects. Concerning pathological patients, Q-SYMBIO represents one of the most important multinational prospective, randomized, double-blind trials performed to evaluate the role of ubiquinol supplementation (100 mg, 3 times daily for 2 years) in 420 patients with moderate to severe heart failure (HF). Although no significant differences were found in primary short-term endpoints at 16 weeks (NYHA functional class, 6 min walk test, and N-terminal pro–B-type natriuretic peptide), long-term endpoints were significantly lower in the CoQ group compared with the placebo one, in particular in terms of cardiovascular mortality (9% vs. 16%, *p* = 0.026), all-cause mortality (10% vs. 18%, *p* = 0.018), and incidence of hospital stays for HF (*p* = 0.03) [43]. Moreover, since geographical differences in patient characteristics and management can affect the outcomes in HF trials, Mortensen et al. [44] assessed the consistency of the treatment effect of CoQ in the European sub-population of 231 patients, obtaining results that confirmed the efficacy of ubiquinol demonstrated in the previous study. Similarly, in a double-blind study involving 102 HF patients, Zhao et al. demonstrated that the use of 30 mg/day of CoQ for 12 months as adjuvant treatment was able to significantly attenuate the incidence of atrial fibrillation compared to placebo group (6.3% vs. 22.2%, *p* = 0.02) [45]. The role of CoQ in the cardiovascular health has also been demonstrated in subjects without CVD manifestations. Alehagen et al. [46], in a 10-year prospective randomized, double-blind trial, evaluated cardiovascular mortality in 443 healthy elderly Swedish subjects supplemented with 200 mg/day of CoQ and 200 µg/day selenium or with placebo over a period of 4 years. In the treated group, a reduced risk of cardiovascular mortality by 50% was observed not only during the intervention period, but this persisted for the duration of the follow-up period. The ability of CoQ to counteract CVD-related complications is mainly due to its bioenergetic and antioxidant roles and its efficacy strictly depends on many factors, such as the dosage and duration of supplementation. In 2012, Lee et al. [47] found significantly lower plasma levels of CoQ in subjects with coronary artery disease (CAD) compared to the control group (healthy individuals with normal blood biochemistry) and subjects with CoQ plasma levels that are higher than 516 nmol/L showed a significantly lower risk of CAD. By supplementing with 150 mg/day of CoQ for 8 weeks, the same authors showed a decrease in MDA levels while, if the supplementation was extended to 12 weeks, a significant increase in antioxidant enzyme activities (catalase and superoxide dismutase) in patients with CAD occurred [48]. Finally, patients with CAD during statin therapy benefited from supplementation with 300 mg/day of CoQ for 12 months in terms of both the enhancement of antioxidant enzymes activities and lowering of inflammatory markers [49]. Although the role of CoQ on counteracting the inflammatory response has been widely demonstrated, in our recent study, supplementation with 400 mg/day of ubiquinol from 7 days before to 5 days after aortic valve replacement in 50 elderly patients affected by severe aortic stenosis, was not able to curb the inflammatory response triggered by cardiac surgery procedure [50]. This was probably due to a remarkably high inflammatory state (plasma IL-6 < 100 pg/mL) associated with the post-surgical intervention in this cohort, characterized by elderly patients. In fact, studies supporting anti-inflammatory activity of CoQ in vivo are associated with lower levels of inflammation [49,51,52]. Preclinical evidence on the role of CoQ in improving endothelial functionality, previously reported, have also recently been confirmed in clinical studies. Endothelial dysfunction is an early disease marker, associated with different forms of CVD, such as hypertension, coronary artery disease, chronic heart failure, peripheral artery disease, diabetes and chronic renal failure. This is characterized by a reduced endothelial vasodilator response, mainly caused by a low NO bioavailability and increased level of ROS [53]. Many studies highlighted the ability of both ubiquinone and ubiquinol to counteract endothelial impairment in CVD patients. In a randomized, double-blind, placebo-controlled trial, the supplementation with 300 mg/day of CoQ for 8 weeks significantly improved endothelial function in 28 patients with ischemic left ventricular systolic dysfunction compared to placebo [54]. Similarly, 14 patients with chronic heart failure with reduced ejection fraction benefited from improved endothelial functionality after supplementation with 400 mg/day of ubiquinol for 3 months [55]. In a study by Perez-Sanchez et al. [56] in addition to a decrease in pro-thrombotic and pro-inflammatory mediators, an increase in mitochondrial size and an upregulation of genes related to mitochondrial biogenesis, the authors also observed an enhancement in endothelial function in 36 patients with antiphospholipid syndrome, supplemented with 200 mg/day of ubiquinol for 1 month. In contrast, although there are few clinical trials, there is no evidence supporting the ability of ubiquinone to improve endothelial functionality in healthy subjects. Raitakari et al. [57] showed that 150 mg/day of CoQ for 4 weeks did not significantly increase the flow-mediated dilation (FMD) of the brachial artery (4.3% vs. 5.1%, *p* = 0.99) in healthy hypercholesterolemic subjects. Conversely, our recent study highlighted the protective effect of ubiquinol towards endothelial function in subjects with mild-to-moderate dyslipidemia and with no other evidence of disease [58]. In this randomized, double-blind study, 51 subjects with mild-to-moderate dyslipidemia and no clinical signs of CVD were randomized to receive either ubiquinol (200 mg or 100 mg/day) or placebo for 8 weeks. The baseline FMD values were found to be comparable to those observed in the Raitakari population study and, in both treated groups, a significant increase in this parameter (200 mg/day = +1.28%; 100 mg/day = +1.34%) was observed. This could be due to the significantly higher bioavailability of orally administrated ubiquinol, compared with ubiquinone and its direct antioxidant activity [59].

## 3. Fertility

Infertility, defined as impossibility to conceive a child after more than one year of unprotected sexual activity, is a common and emerging burden affecting 50 to 80 million individuals [60]. Causes of infertility are heterogeneous and interest both male and female partners. In this respect, in otherwise healthy subjects, in the absence of organic dysfunction, male infertility is often associated with altered number, motility and morphology of spermatozoa (astenozoospermia); similarly female infertility is often related to the general quality of oocytes [61]. Oxidative stress, often associated with metabolic dysfunction and ageing, is a major factor influencing the qualitative parameters of gametes [62,63,64] and, accordingly, the therapeutic use of antioxidants is a common strategy aiming to increase the fertility rate [64]. Among antioxidant interventions, an important body of literature already reviewed in Littarru et al., 2010 [2], supports the efficacy of CoQ both in improving sperm quality in terms of count and motility, correlated to increasing Q10 levels in spermatozoa. This evidence was confirmed in recent studies showing that ubiquinone [65,66] and ubiquinol [67,68] use in men affected by idiopathic oligoasthenozoospermia, led to improved sperm count, motility and morphology, associated with an increase in spermatic antioxidant defenses quantified as superoxide dismutase (SOD), catalase (CAT), glutathione peroxidase (GPx) levels and sperm DNA integrity [65,66]. Moreover, Tirabassi et al. [69] demonstrated that 3 months of 200 mg/day CoQ intervention, combined with 2.66 g/day aspartic acid, on idiopathic asthenozoospermia patients, increased CoQ (*p* < 0.001) and aspartic acid (*p* = 0.022) levels in sperm cells and seminal fluids, and were associated with significant increases in SOD activity and a significant decrease in oxidative stress markers (NO and DNA damage), the latter showing an inverse correlation with CoQ content. Overall, in this experimental setting, antioxidant protection was associated with an increase in sperm motility (*p* < 0.001). Additionally, Safarinejad et al. [70], in an open label prospective study, showed that CoQ supplementation (300 mg/day for one year), led to a highly significant increase in sperm motility, density, and morphology, and was associated with a 34% total pregnancy rate over 6 months. However, a strong limitation of this study was the lack of a control group. Gvozdjáková et al. [71] reported the effect of ubiquinol in association with other antioxidants (440 mg l-carnitine fumarate, 30 mg ubiquinol, 75 IU vitamin E, 12 mg vitamin C) up to 6 months of treatment. They observed that an increase in antioxidant content in seminal fluid was associated with a significant improvement in spermatozoa kinetic parameters and a concomitant increase in 45% pregnancy rate. Finally, Kobori et al. [72] reported that 6 months of treatment with CoQ (120 mg/day) and vitamin E and C (80 and 40 mg < 7 day) led to a significant increase in sperm count and motility as well as pregnancies (48 out of 169 patients) either natural or assisted with reproductive technology. In relation to female fertility concerning oocyte quality, CoQ intervention was considered in relation to poor ovarian reserve capacity, altered oxidative status, associated with metabolic imbalances, and antioxidant deficiency in follicular fluids. Giannubilo et al. [73] showed that ubiquinone oral supplementation (200 mg/day for 1 month) led to an increase in follicular CoQ content, but antioxidant capacity measured through ORAC assay was lower in CoQ-supplemented mature oocytes compared to untreated ones. This counter-intuitive behavior of antioxidant capacity was interpreted by authors in light of a dysfunctional blood-follicular barrier in mature subjects, leading to altered follicular fluid composition and potential compensatory effects counteracted by CoQ, providing a preservation of follicular homeostasis. Concerning in vitro fertilization technology, a large retrospective study conducted by Gat et al. [74] on women with poor ovarian reserve capacity and subjected to in vitro fertilization (IVF) or controlled ovarian hyperstimulation plus intrauterine insemination (IUI), showed that the combined treatment of CoQ (600 mg per day) with dehydroepiandrosterone (DHEA), a steroid hormone (75 mg per day), was superior in comparison with DHEA supplementation alone in increasing pre-stimulation parameters in IVF and IUI cycles, such as follicular antral count (AFC) (*p* = 0.0001), mature follicule number (*p* = 0.01) and gonadotropin dose (*p* = 0.003). Similarly, Xu et al. [75] showed in a study involving 186 poor ovarian reserve capacity women supplemented with CoQ before in vitro fertilization procedure, that there was a significant increase in high quality embryos (*p* = 0.03), and estradiol production (*p* = 0.02). In this case, too, patients required a lower gonadotropin induction dose. Finally, Bentov et al. [76] in a double-blind placebo controlled randomized trial that included 27 in vitro fertilization-intra cytoplasmic sperm injection (IVF-ICSI) patients, 35–43 years of age, taking either 600 mg of CoQ or placebo, did not observe any differences in the post-meiotic aneuploidy rate; however, as declared by the authors, the study was underpowered. Besides human studies, animal models have provided additional evidence of the efficacy of CoQ and have led to some important breakthroughs in terms of mechanistic interpretation of its beneficial effects. In particular, in male Sprague Dawley rats exposed to a single dose of ionizing radiation (10 gray), oral treatment with ubiquinone mg/kg for 2 weeks, was able to prevent radiation-induced histological aberration and mitochondria pro-apoptotic signaling in testes (*p* < 0.05), suggesting a role of CoQ in preventing mitochondria dysfunction and its implication in testicular toxicity [77]. More evidence has been collected on models of ovarian dysfunction using cyclophosphamide-treated rats, which showed that CoQ intervention (22 mg/kg per day for 21 days) increased the number and quality of oocytes, as well as embryo development stages, by modulating the expression of genes involved in folliculogensis, such as proliferating cell nuclear antigen (PCNA) (*p* < 0.05) and follicle stimulating hormone receptor (FSR) (*p* < 0.05) [78]. Oxidative stress, in particular in females, is heavily involved with reproductive ageing, that notably has an early onset compared to other vital functions. In this respect, Zhang et al. [79] showed the effect of CoQ in improving post-ovulatory aged oocytes in mice, by suppressing markers of oxidative damage while increasing the amount and physiological distribution of juno and ovastacin, proteins with a critical function in the fertilization process, associated with sperm binding activities. Similarly, Niu et al. [80] highlighted the promising effect of ubiquinol in rescuing post-ovulatory aging in cultured porcine oocytes, through the induction of proteins involved in mitochondrial biogenesis and mitophagy, such as SIRT1 and PGC1α at mRNA level and Pink1 and Parkin 1 at the protein level. Regarding reproductive aging in animals, Ben Meir et al. [81] reported that ubiquinone injected in mice at 22 mg/kg for a period of least 12 weeks, was able to counteract oocyte aging in terms of ovarian reserve capacity (*p* = 0.01) and oocyte quality (*p* = 0.05) in old mice. The novel efficacy mechanism of CoQ was related to an improvement in mitochondrial oocyte functionality parameters, such as active mitochondria pool (*p* < 0.001), flavin adenine dinucleotide (FAD) reduction capacity (*p* < 0.001) and membrane potential (*p* < 0.05) in aged oocytes.

## 4. Muscle Health and Physical Exercise

The health benefits of moderate and regular physical exercise are well supported by the scientific literature. On the other hand, strenuous physical exercise is associated with detrimental effects due to an increase in oxygen consumption up to 100-fold higher than under resting conditions in the skeletal muscle and a consequent rise in reactive oxygen species (ROS) [82]. Previously, we reviewed several pieces of evidence supporting the role of CoQ, related mainly to ubiquinone interventions, in improving aerobic capacity in terms of maximal oxygen consumption and fatigue sensation. These outcomes are mainly related to its capacity to attenuate oxidative stress and pro-inflammatory signaling, to lowering levels of plasmatic markers of muscular damage (creatine kinase and myoglobin) as well as to decreasing lipid peroxide exercise, induced in athletes [2]. In the last decade, more evidence has also been gathered in relation to the use of the reduced form, ubiquinol, which chemically is the active form as an antioxidant. Under physiological conditions, both total CoQ content and its oxidative status, could be influenced, in particular by aging, nutrition, drug use [83] and certainly by intense physical activity [84]. In relation to the latter, according to a study by Doring et al. [85], analyzing CoQ as a determinant of muscular strength in two independent cohort studies on 1301 subjects, the authors concluded that both low CoQ/cholesterol levels and the percentage of the reduced form of CoQ, represent indicators of an increased risk of age-associated muscle weakness and reduced muscle mass in humans due to their negative associations with upper body muscle strength, peak flow and muscle mass. Sarcopenia, defined as a decrease in muscle quantity and quality [86], is a frequent disease condition associated with aging, often characterized at the cellular level with structural and functional alterations in skeletal muscle mitochondria. In this context, the association of mild regular physical exercise and ubiquinol supplementation, as a mitochondrial nutrient, has been proposed as a strategy to slow down skeletal muscle senescence and improve the quality of life in the elderly. In this respect, in a murine model of accelerated ageing, SAMP8 mice [87] showed that a 2 month program of physical exercise in this animal model significantly increased mitochondrial muscle damage, but the combination with ubiquinol supplementation was able to preserve mitochondrial ultrastructure, promoting mitochondrial biogenesis and counteracting the deleterious effects of physical exercise-derived ROS in the senescence-prone oxidative stress mouse model. Besides sarcopenia, the muscles of elderly people are at higher risk of developing myopathy as a side effect of statin treatment [88] widely used cholesterol-lowering drugs. In severe cases, statin-associated damage may even lead to rhabdomyolysis. In fact, statins, by acting as selective inhibitors of HMG-CoA reductase, an enzyme that catalyzes an early step of the mevalonate pathway, lower both cholesterol and CoQ biosynthesis. The molecular mechanisms underlying statin-induced myotoxicity are not well established. Several studies propose that statins promote cell death, inducing mitochondrial dysfunction. Another hypothesis suggests that myotoxicity is mediated by inhibition of mitochondrial respiratory chain as a consequence of CoQ depletion. Animal model studies have been used to support a protective effect of CoQ in association with statin treatment. In particular, an ex vivo rat study showed that 1 h co-incubation of muscle fibers obtained from soleus biopsies with increasing concentrations of simvastatin (1-40 μM) and 1 mM L-carnitine, 100 μM mevalonate or 10 μM CoQ, prevented statin toxicity, supporting mitochondrial respiration and protecting against hydrogen peroxide generation [89]. Accordingly, in humans, Wang LW et al. [90] reported that CoQ facilitated the recovery of patients affected by statin-induced skeletal muscle damage associated with pain and weakness. In a human double-blind, randomized, placebo-controlled study involving patients with previous intolerance to statins, 3 months of 100 mg/day of ubiquinone, in addition to statin (half dose compared to the prescribed dose at enrollment), improves the perception of clinical symptoms such as asthenia, myalgia or pain [91]. Some authors hypothesized that muscular mitochondrial dysfunction due to CoQ deficiency induced by statin treatment might influence also physical performance. In fact, some authors have observed that CoQ administration (PureSorb-Q40-CoQ10, a water-soluble form of CoQ; 10 mg/L in drinking water) in atorvastatin-treated mice, reversed atorvastatin-related mitochondrial dysfunction, counteracting statin-induced decrease in oxygen utilization, improving exercise endurance and tolerance [92]. Within exercise under physiological condition, only a limited set of studies have investigated the role of CoQ as an antioxidant supplement in sports nutrition to counteract oxidative stress induced by strenuous exercise. In fact, despite the beneficial effects of moderate and regular physical exercise, strenuous exercise, defined as any activity that expends six metabolic equivalents (METS) per minute or more [93], may become detrimental. In particular, Simon H.B.’s review [94] underscores how repetitive intense exercise may have deleterious cardiovascular effects, causing structural damage to muscle cells, leading to muscle soreness, swelling, prolonged loss of muscle function, increased ROS production, induction of pro-inflammatory signaling, impairment of immune functions and leakage of muscle proteins into circulation [95,96]. In these overtraining conditions, subjected to individual training capacity and fitness level, CoQ supplementation as a mitochondrial nutrient, and in particular ubiquinol, due to its antioxidant activity, may modulate these harmful responses. Indeed, due to its antioxidant function, in addition to its bioenergetic role inside the mitochondria, CoQ has been tested as an anti-fatigue and ergogenic supplement in relation to physiological challenges, promoting exercise performance and supporting metabolism beyond fatigue threshold. However, these effects are highly debated, and the available results are controversial. In animal models it has been shown that ubiquinol supplementation dose-dependently (102.5 mg/kg, 205 mg/kg or 615 mg/kg of body weight) after 28 days reduced serum lactate and creatin kinase levels induced by forelimb grip strength and exhaustive swimming tests, increasing free fatty acid (FFA) and muscle glycogen content, as major energy reservoir during exercise [97]. In humans, 200 mg/day ubiquinol oral supplementation for 2 weeks in healthy and well-trained firemen was able to modulate inflammatory signaling, expression of pro-inflammatory molecules by increasing some anti-inflammatory cytokines during high intensity physical exercise, revealing moreover a possible pro-angiogenic effect [98]. Moreover, in the same study, ubiquinol improved energetic substrate supply and muscle recovery after strenuous exercise. The presently available scientific data highlight that the variability of biological responses following CoQ intervention are influenced by different aspects, including dosage, oxidative status of the supplement, time of supplementation, training level and type of physical exercise approached by users. In fact, in contrast with previously discussed beneficial effects, Bloomer RJ et al. [99] showed that 1 month of supplementation with 300 mg/day of ubiquinol in healthy trained individuals, increased total and reduced blood CoQ but it was not effective in improving exercise performance nor in lowering oxidative stress markers, after a repeated 5-cycle sprints test. Accordingly, Orlando P. et al. [84] confirmed these results, showing that 1 month of 200 mg/day of ubiquinol supplementation minimized exercise-induced CoQ depletion, enhancing plasma and cellular antioxidant levels, but this was not able to improve physical performance indexes or markers of muscular damage, after a single bout of intense exercise (40 min run at 85% of maximal heart rate). However, according to Gul I et al. [100], supplementation with ubiquinone 100 mg/day for 8 weeks partially prevented the increase in lipid peroxidation after repeated short-term supramaximal exercise in healthy sedentary men, emphasizing the fact that CoQ supplementation is more efficient when associated with moderate and regular physical exercise.

## 5. Other Clinical Aspects

Due to its ubiquitous distribution, its pivotal role in mitochondrial bioenergetics and its antioxidant role in biological membranes, wider clinical applications of CoQ continue to attract attention in recent research. As discussed earlier, being cardiovascular health a major field of application of CoQ, it represents a candidate molecule for treatment of systemic pathologies that significantly overlap with cardiovascular dysfunction, either as a cause or a consequence, for instance metabolic diseases and neurodegeneration. Considering these vital functions, CoQ deficiency represents a critical clinical aspect. CoQ deficiency can result from either a genetic defect in its biosynthetic pathway, known as primary CoQ deficiency, or it can occur as a result of diseases not associated with this or from certain pharmacotherapies, and is known as secondary CoQ deficiency [101,102]. Primary CoQ deficiency is an autosomal recessive condition, and to date, mutations in 10 genes have been associated with this condition: PDSS1, PDSS2, COQ2, COQ4, COQ5, COQ6, COQ7, COQ8A, COQ8B, and COQ9 [103]. The clinical presentation of primary CoQ deficiency is extremely heterogeneous and although there has been some attempt to divide it up into five distinct clinical phenotypes: encephalomyopathy, severe infantile multisystemic disease, steroid resistant nephrotic syndrome, cerebellar ataxia and isolated myopathy, other clinical presentations have also been associated with CoQ deficiency [101,104]. At present, the incidence of primary CoQ deficiency is estimated to be below 1:100,000, although no precise epidemiologic data are available, and this condition is thought to be underdiagnosed since the assessment of endogenous CoQ status is only available at specialist clinical centers [104]. In contrast to primary CoQ deficiencies, the incidence of secondary CoQ deficiencies is thought to be relatively common and most recently has been associated with disorders, such as phenylketonuria (PKU) and the lysosomal storage disorder, mucopolysaccharidosis Type III [105]. The actual mechanisms responsible for secondary CoQ deficiencies in disease is uncertain, although oxidative stress induced catabolism of CoQ together with the inhibition of the enzymes involved in its synthesis have been suggested [102,106]. As discussed in the muscle health section, HMG-CoA reductase inhibitors (statins) have been associated with CoQ deficiency and this has been suggested as a possible contributing factor to the myopathic side effects associated with this pharmacotherapy [107]. Once biochemical evidence of a CoQ deficiency has been detected, further studies are required to determine whether it is a primary or secondary CoQ deficiency [107]. The former determination requires next generation sequencing diagnostic strategies and the latter may require radiolabelled incorporation studies [107]. Once diagnosed, patients with CoQ deficiency respond well to high dose oral CoQ supplementation, although early diagnosis and treatment is essential to ensure an optimal clinical response [104]. Whilst CoQ supplementation appears to improve peripheral abnormalities, neurological symptoms appear to be more refractory to treatment and this may reflect the limited ability of CoQ to cross the blood–brain barrier [108]. A deficit in CoQ status has been reported in neurological disorders, resulting in antioxidant status disturbances and mitochondrial dysfunction frequently leading to severe neuronal degeneration [109,110]. Therefore, CoQ supplementation has been extensively used as a treatment in neurodegenerative diseases, including Alzheimer’s disease (AD), Parkinson’s disease (PD) and Multiple Sclerosis (MS) where it has been shown to exert neuroprotective effects against oxidative stress-induced damage and mitochondrial respiratory chain (MRC) dysfunction. Experimental studies have suggested CoQ as a potential therapeutic candidate, preventing the harmful effects of β-amyloid (Aβ) deposition on human endothelial cells in early asymptomatic stages of AD, therefore, delaying the progression of severe neuropathology [111]. Pre-treatment with CoQ prevented Aβ-induced cellular toxicity and oxidative injury by inhibiting Aβ trafficking and accumulation into the mitochondria, cytochrome c release and mitochondrial permeability transition pore (mPTP) opening, and superoxide anion (O_2_^•-^) and hydrogen peroxide (H_2_O_2_) production. The presence of Aβ significantly elevates serum MDA and total oxidant levels, in addition to a reduction in antioxidant enzyme activity, including SOD, GPx and CAT, resulting in impaired synaptic plasticity in a rat model of AD [112]. CoQ supplementation was found to significantly reverse these consequences, suggesting that treatment with CoQ can prevent the harmful effects of Aβ on hippocampal synaptic plasticity via increasing the total antioxidant capacity (TAC) levels. Alzheimer type dementia is a characteristic feature of Down Syndrome (DS) patients associated with increased oxidative stress and premature senescence. A lower plasma content of CoQ was observed in young patients with DS compared to healthy subjects of the same age and is positively correlated with the intelligence quotient, suggesting a role of this molecule in neurodevelopment [113]. CoQ intervention in DS children was conducted within a 5-year intervention trial and, despite previous promising results [2], supplementation with 4 mg/kg per day of CoQ in the oxidized form led to no effects on DNA oxidation at both short (6 months) [114] and long-term treatment (4 years) [115]. Nonetheless, as pointed out by the authors, both the therapeutic dosage and the form of CoQ used were not ideal for this intervention. In fact, Miles et al., 2007 [116] have shown that 10 mg/kg of ubiquinol was able to significantly improve oxidative imbalance in DS patients. In other neurodegenerative disorders, such as PD, reduced plasma levels of total CoQ and elevated ratios of oxidized CoQ/total CoQ have been reported, suggesting that CoQ oxidation may have a pathogenic role in PD [117]. In a PD rat model, the intrastriatal distribution of CoQ was demonstrated to inhibit apoptosis, resulting in an elevation of factors responsible for neuronal growth and enhanced angiogenesis, suppressing neurodegeneration and preventing the progression of PD [118]. In MS, CoQ-treated patients (500 mg/day) were found to have significant elevations in SOD activity and plasma total antioxidant capacity (TAC) and reductions in MDA levels in comparison to the placebo control group, suggesting that CoQ can increase the activity of antioxidant enzymes and reduce oxidative stress in patients with MS [119]. However, patients with a CoQ deficiency often show inconsistent responses to oral CoQ supplementation, with neurological disease patients demonstrating the highest percentage of ineffective results [108,120]. Poor uptake of CoQ treatment in neurodegenerative disease may be due to its low oral bioavailability, poor solubility and transfer across the blood–brain barrier, which may compromise its effectiveness as a treatment for neurodegenerative disorders. The development of CoQ-loaded nanoemulsions have been studied in order to improve the oral bioavailability of CoQ supplementation in rat models of PD, which was shown to reduce nigrostriatal dopamine depletion, significantly improving behavioral activity in comparison to control rats treated with a CoQ suspension [121]. In addition, reduced elevated levels of thiobarbituric acid reactive substances (TBARs) and increased glutathione levels were reported. In a mouse model of mitochondrial encephalopathy (Coq9X/X), oral ubiquinol-10 supplementation—a water-soluble, reduced form of CoQ—was found to increase CoQ concentrations in the cerebrum of Coq9X/X mice, demonstrating that ubiquinol-10 may be more effective at increasing CoQ-dependent MRC complex activities as well as reducing cerebral oxidative damage, therefore improving the efficacy of CoQ therapy in neurodegenerative diseases [120]. Therefore, these findings suggest that CoQ adjuvant treatments can enhance the bioavailability and antioxidant properties of CoQ, therefore providing a promising treatment for neurodegenerative disorders. Another important field of application is represented by diabetes, and its cardiovascular and neuropathic complications. Diabetic neuropathy is the main complication of diabetes. The long-term administration of CoQ in diabetic rat [122] and mouse [123,124] models decreases oxidative stress and lipid peroxidation, increasing TAC and glutathione (GSH) levels, modulates the expression of caspase 3 and UCP2 proteins, and reduces proinflammatory factors, resulting in a reduction in atrophy and loss of dorsal root ganglion (DRG) neurons, as well as improvement of motor function and neuropathic pain-downregulating phospholipase C β3. These effects are potentiated by CoQ and alpha-lipoic acid (ALA) co-treatment [122]. CoQ administration inhibits other diabetic complications such as glomerulosclerosis and leukocyte infiltration [125]. This could be due to the protection of endothelial cells from apoptosis and dysfunction induced by high glucose through the AMPK pathway [126]. In murine glomerular endothelial cells (mGECs), CoQ protected against diabetic nephropathy-induced mitochondrial dysfunction via mitophagy by restoring Nrf2/ARE signaling [127]. Moreover, in murine cross-generational models of insulin resistance in the offspring, associated with poor maternal nutrition, post-weaning CoQ supplementation has been shown to have anti-inflammatory properties and to modulate insulin-signaling protein expression, thus preventing the development of insulin resistance associated with rapid post-natal growth [128]. Finally, CoQ intervention has also been tested in relation to nephrological and hepatological dysfunction. In this respect, there are conflicting data on the effects of CoQ in kidney disease. In a gentamycin-induced nephrotoxicity rat model, CoQ effect was mild and limited to a lower rate of tubular necrosis and hyalen accumulation [129]. On the contrary, in patients with lithiasis undergoing extracorporeal shockwave lithotripsy, CoQ improved renal injury increasing glomerular filtration and decreasing albumin/creatinine ratio and β2-microglobulin level, in addition to the improvement in vasoactive molecules (renin and aldosterone) and in pro-inflammatory mediator levels. However, these changes were not associated with enhancement in oxidative stress parameters [130]. In haemodialysis patients, CoQ plasma level was positively correlated with brachial artery flow-mediated dilatation and negatively correlated with oxidative markers (MDA and 8-hydroxydeoxyguanosine) suggesting that low CoQ levels could contribute to endothelial dysfunction in these patients [131]. Finally, CoQ through its antioxidant and anti-inflammatory properties, was found to have positive effects on biochemical parameters related to various pathologies, such as non-alcoholic fatty liver disease (NAFLD) [132], and to protect against the side effects of anticancer therapy, such as radiotherapy-induced gastrointestinal injury in patients with pelvic tumors [133] and doxorubicin (DOX)-induced testicular toxicity [134].

## 6. Emerging Applications of Coenzyme Q_10_

Two emerging areas of investigation of CoQ in health in the last decade refer to ophthalmological interventions and skin ageing research.

Retinal disease, such as age-related macular degeneration, diabetic retinopathy, retinitis pigmentosa and glaucoma, are the major causes of blindness in the world and are characterized by oxidative stress. In the retina, high oxygen consumption results in the production of large amounts of ROS that could promote cellular damage and, subsequently, retinal degeneration. Due to its antioxidant properties, CoQ topical eye preparations have demonstrated potential beneficial effects for patients with retinal disease. In particular, CoQ has been reported to have beneficial effects on glaucoma, a multifactorial neurodegenerative disorder during which oxidative stress activates astrocytes that are involved in optic nerve head (ONH) remodeling, associated with the pathogenesis of glaucoma. In rat ONH astrocytes exposed to H_2_O_2_, CoQ treatment prevented cell activation, decreasing SOD2 and hemeoxygenase-1 (HO–1) protein expression, and loss of mitochondrial mass. In addition, CoQ counteracted the decrease in cell viability and ATP and the increment in ROS production [135]. Similar results in improving retinal ganglion cell degeneration by preventing oxidative stress, mitochondrial alterations and astroglial activation were observed in glaucomatous DBA/2J mice fed with CoQ (1% *v/v*) for 6 months [136]. While open-angle glaucoma (OAG) patients treated with eye drops containing CoQ and vitamin E (in addition to a β-blocker monotherapy) for 12 months showed beneficial effects on functions of the inner retina and enhanced visual cortical response [137]. Moreover, the combination of two antioxidants, CoQ and melatonin, and an anti-inflammatory drug, dexamethasone, incorporated in a novel multi-loaded poly lactic-co-glycolic acid (PLGA)-microspheres, has been proposed as a novel neuroprotective intraocular therapy for the treatment of glaucoma [138]. CoQ in ophthalmic solution has also been shown to reduce corneal damage associated with UVB exposure in both in vitro and in vivo models. In UVB-exposed human corneal cells, CoQ supplementation led to an increased survival rate and mitochondrial function counteracting ATP decline. In a rabbit model, treatment with CoQ eye drops (2 per eye every 8 h for 3 days) reduced UVB-induced vessel hyperemia and promoted corneal wound healing after corneal epithelium removal [139]. CoQ, due to its antioxidant properties, protected ganglion cell death in mouse ischemic retina, decreasing oxidative stress and counteracting the increased expression of apoptosis-associated protein Bax and activation of both astroglial and microglial cells, thus decreasing the expression of glial fibrillary acidic protein and ionized calcium-binding adapter molecule 1 (Iba-1) [140]. In humans, CoQ oral supplementation (100 mg per day), in combination with vitamins, has also been shown to improve the visual field in retinal dysfunction caused by vascular disorders, such as non-arteritic ischemic optic neuropathy, retinal artery occlusion and hemianopia/quadrantanopia [141]. In conclusion, CoQ has demonstrated protective effects on different retinal diseases by decreasing oxidative stress, counteracting microglia cell activation and maintaining mitochondrial functionality, thus resulting a key molecule in eye health.

Skin is the largest organ of the body which directly interfaces with the external environment, and it is therefore constantly challenged by environmental factors, such as ultraviolet (UV) radiation, smoking and pollution, all of which contribute to extrinsic aging of skin, in contrast to intrinsic aging due to genetic factors [142]. In the skin, CoQ is found in both cells of the human dermis and epidermis where, in the latter, its concentration is 10-fold higher [143] and where approximately 46% of total CoQ is present in its reduced form [144]. It is also a constituent of skin surface lipids (SSL) of the stratum corneum (SC). Previous studies prior to 2010 have shown that CoQ levels in skin and SSL decline with age and with UV exposure [145,146,147] and this has been supported by more recent advancements in the understanding of the role of CoQ in skin aging. Marcheggiani et al. [148] demonstrated, in cultured human dermal fibroblasts (HDF), that the depletion of CoQ is not a consequence but a cause of skin aging. Furthermore, in the same study, supplementation with ubiquinol led to increased bioavailability, compared to its oxidized form in a senescence model of skin ageing, and was shown to be more efficient in rescuing the senescent phenotype both at the cellular and mitochondrial levels [149]. These results appear to reinforce the role that CoQ has in minimizing skin aging and are in line with the work from Zmitek et al. conducted on human volunteers to demonstrate the important role that dietary intake of CoQ has on improving skin structural features associated with skin aging (mimic wrinkles/poor skin tone/visual dryness). On a cohort of 34 healthy female volunteers aged between 45–65 years, those receiving an investigational product for 12 weeks, based on a liquid food supplement, characterized by a combination of water-soluble CoQ (Q10Vital**^®^**, 50 mg) and fish collagen (4.0 g), reported better skin parameters compared to placebo controls [150]. However, no significant effects of the supplementation on skin hydration, dermis thickness, transepidermal water loss (TEWL) and viscoelasticity were observed. This was in accordance with a previous study on a cohort of 33 female volunteers [151] where in those given the same liquid food supplement but containing only CoQ (either 50 or 150 mg/day for 12 weeks), a significant reduction in periorbital wrinkles, microrelief lines, and improved skin smoothness with both doses was seen, whereas the higher dose led to additional improvement of wrinkles in other facial parts. Further beneficial effects of CoQ on skin aging have also surfaced with the advent of new technologies, such as the Seahorse XF24 extracellular flux analyzer. With this method, Schniertshauer et al. demonstrated that mitochondrial respiration and ATP production in epithelial tissue derived from human skin biopsies (male and female donors, 29–84 years, undergoing general or plastic surgery) decreased with donor age (approximately 10%/decade). Respiratory parameters were restored when the reduced form of CoQ, ubiquinol, was administered as a formulation (QuinoMit Q10 fluid, 100 μM) to the overnight-separation media. The authors concluded that these effects were mainly caused by an increase in the electron transport chain [152]. Indeed, exogenously added CoQ appears to act as an “electron clamp” enhancing electron transport between complexes I/II and III in the mitochondrial respiratory chain, which becomes “leaky” over the years. This study confirmed earlier work by Knott et al., where the seahorse system showed that treatment with 18 µM ubiquinone, a quantity comparable to that found within the human epidermis after 2 weeks of topical application of a test formula containing 870 µM ubiquinone, significantly stimulated energy metabolism in human cultured keratinocytes. Furthermore, these same authors showed that the application of ubiquinone on 73 healthy, non-smoking, female volunteers (20–66 years) led to a significant increase in ubiquinol in human epidermis, thus indicating effective supplementation and transformation. Additionally, they demonstrated that topical CoQ was capable of improving the antioxidant properties of stressed skin [153]. Obviously, effective topical delivery depends on the delivery system used, and several studies in recent years have investigated this aspect, especially with regard to CoQ delivered by nano-structured lipid carriers (NLC), innovative carrier systems derived from oil/water (*o*/*w*) emulsions. Indeed, the first NLC-containing cream on the cosmetic market was introduced in 2005 and it contained ubiquinone, the Cutanova Nanorepair Q10 cream (Dr. Rimpler GmbH, Wedemark, Germany). Pardeike et al. [143] investigated this cream containing 0.48% ubiquinone with an identical *o*/*w* emulsion containing no NLC but the same concentration of ubiquinone. They observed on a cohort of 50 volunteers (22–57 years), that skin hydration and subjective feeling was significantly higher with the Nanorepair Q10 cream. Yue et al. [154] showed that CoQ incorporated in NLC, based on soybean lecithin, efficiently protected HDF against UVA-induced oxidative damage and restored SOD and glutathione peroxidase activities compared to a CoQ emulsion. Furthermore, the skin penetration capability of CoQ-NLC versus CoQ-emulsion was greater. This study enforces the role that NLC-based formulations could have an ineffective topical drug delivery of CoQ. Bruge et al. [155] later compared the efficacy of NLC loaded with either the oxidized or the reduced forms of CoQ against UVA-mediated damage in HDF and found that only reduced CoQ-NLC was efficient in counteracting UVA-associated mitochondrial depolarization, suggesting a potential role of this molecule in anti-aging cosmetic formulations. Nanoemulsions (NE) have also been explored to optimize topical skin permeability of CoQ. El-Leithy et al. [156] tested the solubility of CoQ in various oils, surfactants, and co-surfactants and came up with an optimized formula consisting of 10% *w*/*w* isopropyl myristate (oil phase), 60% *w*/*w* Tween 80:Transcutol HP mixture at a ratio 2:1, 30% *w*/*w* water and 2% *w*/*w* ubiquinone. When tested on the dorsal skin of 12–18-month-old female rats, applied twice daily for 1 month, a reduction in skin wrinkles and smoother skin compared to the control emulsion devoid of CoQ was observed. Besides CoQ’s role as an antioxidant and energizer in skin, recent studies point to its possible role as anti-inflammatory and skin-whitening agent. Zhang et al. [157] observed that CoQ (0.5–2 µM) dose-dependently reduced melanin synthesis by inhibiting tyrosinase activity in melanoma B16 cells, which points to the potential depigmentation effect of CoQ in skincare products. It also decreased IL-1α expression in UVB-irradiated HaCaTs, suggesting an anti-inflammatory function of CoQ. Furthermore, CoQ dose-dependently (0.5–10 µM) enhanced elastin and collagen IV gene expression, as well as cell proliferation in adult fibroblasts, and inhibited ROS production and MMP-1 content in both UVA/B-irradiated fibroblasts and HaCaTs.

## 7. Concluding Remarks

**Figure 1:**
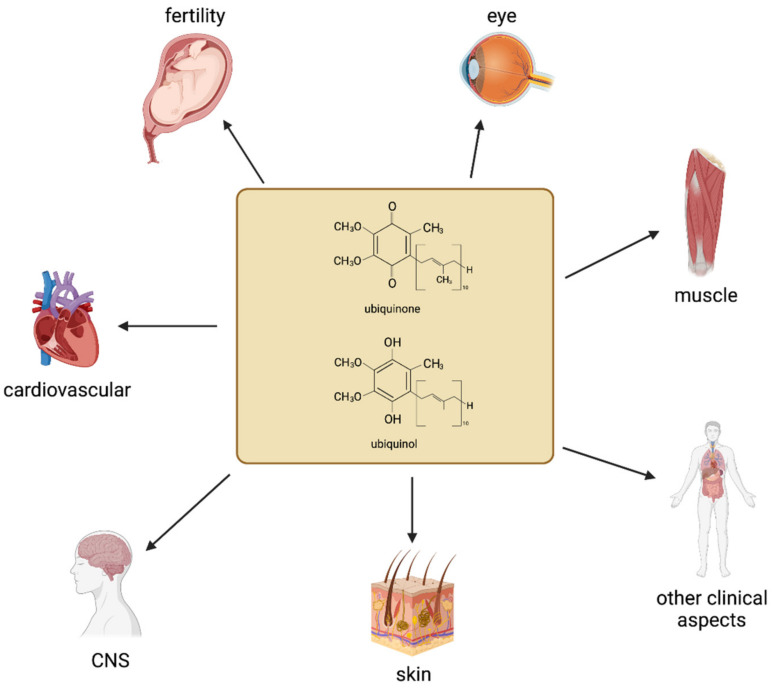
Areas of CoQ_10_ application in human studies for improving human health. CNS = Central Nervous System.

In the second decade of the new millennium, 64 years after its discovery, CoQ research is still attracting a lot of interest in the scientific community, both in relation to its multifaceted mechanism of action and for its wide range of applications in human health (Figure 1). In terms of research article production, reviewed here, the major areas of intervention remain cardiovascular health and human fertility. In these studies, the beneficial effects of CoQ mainly rely on its well-known bioenergetic and antioxidant functions, although novel interactions with cellular biochemical pathways through redox signaling and modulation of mitochondrial function are progressively being described. Limited bioavailability of CoQ remains a major issue for its use, although novel formulation strategies and the direct use of ubiquinol, which does not require cellular reduction and is more available to cellular compartments, are highly investigated. In this respect, topical application where the reduced form is particularly advantageous over conventional ubiquinone, represents an emerging area of interest, encompassing both dermatological and ophthalmological applications.

**Table 1:** Clinical evidence on the impact of CoQ supplementation on cardiovascular disease-related outcomes.

Author, Year	Country	Participants	CoQ_10_ Dosage and Duration	Main Findings
[158] Kurban et al., 2010	Turkey	Healthy volunteers	Received 100 or 150 mg/day acetylsalicylic acid for 2 months before quantification of total CoQ_10_	Reduced total oxidant status and oxidized low-density lipoprotein (Ox-LDL) concentration without significantly affecting CoQ_10_ levels
[54] Dai et al., 2011	China	Patients with ischemic left ventricular systolic dysfunction (LVSD)	Received 300 mg/day CoQ_10_ (ubiquinone) for 8 weeks	Improved mitochondrial function and flow-mediated dilation (FMD) to enhance endothelial function
[159] Fumagalli et al., 2011	Italy	Patients with chronic heart failure subjected to physical performance	Received 320 mg/day of Q-terclatrate (equivalent to 16 mg of native CoQ_10_) and 340 mg/day of creatine for 8 weeks	Improved exercise tolerance, by enhancing peak oxygen consumption and quality of life
[160] Mikhin et al., 2011	Russia	Patients with hypertension	Received CoQ_10_ in combination with an angiotensin converting enzyme inhibitor enalapril, during a 24 h blood pressure profile	Combination therapy promoted normalization of vascular endothelial function
[161] Toyama et al., 2011	Japan	Hyperlipidemic patients with coronary artery disease on statins	Quantification of total plasma CoQ_10_ (ubiquinol) levels	Atorvastatin and rosuvastatin, when combined with exercise could significantly preserve ubiquinol levels associated with an increase in HDL-c
[162] Young et al., 2011	New Zealand	Patients with phenotypic or genotypic familial hypercholesterolemia on long-term statin	Quantification of total plasma CoQ_10_ levels	Low CoQ_10_ levels are associated with increased arterial stiffness
[163] Brugè et al., 2012	Italy	Healthy subjects	Received 20 mL extra virgin olive oil per day for 2 weeks, followed by 2 weeks of olive oil enriched with 20 mg and 2 more weeks with 40 mg of CoQ_10_ (ubiquinol)	Increased plasma levels of CoQ_10_ were associated with reduced susceptibility of LDL to peroxidation and improved redox status
[164] Larijani et al., 2013	United States	Firefighters	Received 300 mg/tablet aged garlic extract plus 30 mg/tablet CoQ_10_ (ubiquinone) for 1 year	Combination therapy induced beneficial effects on vascular elasticity and endothelial function
[47] Lee et al., 2012	Taiwan	Patients with coronary artery disease (CAD)	Quantification of total plasma CoQ_10_ levels	Higher CoQ_10_ concentration was correlated with a lower risk of CAD, even after adjusting for the risk factors for CAD
[48] Lee et al., 2012	Taiwan	Patients with CAD	Received 60 or 150 mg/day of CoQ_10_ (ubiquinone) for 12 weeks	Higher CoQ_10_ dose (150 mg) reduced oxidative stress parameters like lipid peroxidation (MDA levels) and increased antioxidant enzyme (superoxide dismutase; SOD) activity
[56] Perez-Sanchez et al., 2012	Spain	Patients with antiphospholipid syndrome	Preincubating of purified monocytes with CoQ_10_ for 24 h	Reduced oxidative stress, improved mitochondrial structure, reverted altered mitochondrial membrane potential, including decreasing the expression of tissue factor, VEGF, and Flt1
[49] Lee et al., 2013	Taiwan	Patients with cardiovascular disease on statins	Received 150 mg twice a day or a single dose 300 mg/day CoQ_10_ (ubiquinone) for 12 weeks	High CoQ_10_ plasma levels were consistent with significantly enhanced antioxidant enzymes activities and lower inflammation
[43] Mortensen et al., 2014	Europe	Patients with moderate to severe heart failure	Received 100 mg/3 times a day to total 300 mg/day of CoQ_10_ (ubiquinol) for 2 years	Improved symptoms and reduced major adverse cardiovascular events. Biomarker status included Brain-Natriuretic Peptide (BNP), and long-term Outcome (hospitalizations/mortality)
[165] Sharp et al., 2014	United States	Patients with pulmonary arterial hypertension	Received 100 mg/3 times a day to total 300 mg/day of CoQ_10_ (ubiquinol) for 12 weeks	High plasma CoQ_10_ levels were associated with improved cardiac parameters and mitochondrial synthetic function. Hemoglobin increased and red cell distribution. However, metabolic and redox parameters, including glutathione levels were not affected
[46] Alehagen et al., 2015	Sweden	Healthy elderly individuals	Received 200 mg/day CoQ_10_ (ubiquinone) plus 200 μg/day organic selenium for 48 months to a 10-year follow-up	Significantly reduced cardiovascular mortality, confirmed not at intervention but during 10-year follow-up
[52] Mohseni et al., 2015	Iran	Hyperlipidemic patients with myocardial infarction	Received 200 mg/day CoQ_10_ (ubiquinone) for 12 weeks	Although did not affect total cholesterol, beneficial effects of CoQ_10_ supplementation were related to increased high-density lipoprotein (HDL) levels, and decreased concentrations of (intercellular adhesion molecule-1 (ICAM-1) and IL-6
[51] Bagheri et al., 2015	Iran	Patients with mild hypertension	Received 100 mg/day CoQ_10_ (ubiquinone) for 12 weeks	Beneficial effects of CoQ_10_ were related to decreasing some pro-inflammatory factors, such as IL-6 and high-sensitivity C-reactive protein (hs-CRP), and in increasing adiponectin
[166] Pérez-Sánchez et al., 2017	Spain	Patients with antiphospholipid syndrome	Received 200 mg/day CoQ_10_ (ubiquinol) for 1 month	Improved endothelial function and decreased monocyte expression of prothrombotic and proinflammatory mediators, inhibited phosphorylation of thrombosis-related protein kinases, and decreased peroxides and percentage of monocytes with depolarized mitochondria
[44] Mortensen et al., 2019	Europe	Patients with moderate to severe heart failure	Received 300 mg/day CoQ_10_ (ubiquinone) for 3 months and 2 years	Reduced major adverse cardiovascular events, all-cause mortality, cardiovascular mortality, hospitalization and improvement of symptoms
[55] Kawashima et al., 2020	Japan	Patients with heart failure with reduced ejection fraction	Received 400 mg/day CoQ_10_ (ubiquinol) for 3 months	Significantly improved peripheral endothelial function
[58] Sabbatinelli et al., 2020	Italy	Healthy subjects with moderate dyslipidemia	Received 100 or 200 mg/day CoQ_10_ (ubiquinol) for 8 weeks	Significantly ameliorated dyslipidemia-related endothelial dysfunction. This was in part by improving blood flow through enhancing nitric oxide bioavailability and lowering oxidized low-density lipoprotein cholesterol
[167] Shikh et al., 2020	Russia	Patients with cardiovascular diseases (CVDs)	Effects of atorvastatin, amlodipine and ethoxidol on the endogenous CoQ_10_ plasma concentration	Patients with CVDs treated with various drugs had CoQ_10_ plasma level statistically significantly lower than in practically healthy individuals
[45] Zhao et al., 2015	China	Patients with moderate to severe heart failure (HF	Received 30 mg/day of CoQ_10_ (ubiquinone) for 6 and 12 months	CoQ_10_ induced significant reduction in the level of malondialdehyde and may attenuate the incidence of atrial fibrillation in patients with HF
[50] Orlando et al., 2020	Italy	Patients with aortic stenosis (AS)	Received 400 mg/day of CoQ_10_ (ubiquinol) from 7 days before to 5 days after surgery	Post-operative increase in troponin I was curbed, plasma CoQ_10_ declined and oxidation were counteracted
[59] Langsjoen at al., 2014	United States	Healthy volunteers	Received 200 mg/day of ubiquinone for 4 weeks before and 200 mg/day of ubiquinol for other 4 weeks, after 4 weeks of washout	Plasma total CoQ_10_ and total CoQ_10_/cholesterol ratio were significantly higher after ubiquinol than ubiquinone

## References

[1] Littarru G.P., Tiano L. (2007). Bioenergetic and antioxidant properties of coenzyme Q10: Recent developments. Mol. Biotechnol..

[2] Littarru G.P., Tiano L. (2010). Clinical aspects of coenzyme Q10: An update. Nutrition.

[3] WHO The Top 10 Causes of Death. https://www.who.int/news-room/fact-sheets/detail/the-top-10-causes-of-death.

[4] North B.J., Sinclair D.A. (2012). The intersection between aging and cardiovascular disease. Circ. Res..

[5] Gosse P. (2005). Left ventricular hypertrophy as a predictor of cardiovascular risk. J. Hypertens. Suppl..

[6] Krim S.R., Campbell P., Lavie C.J., Ventura H. (2013). Micronutrients in chronic heart failure. Curr. Heart Fail. Rep..

[7] Gao L., Mao Q., Cao J., Wang Y., Zhou X., Fan L. (2012). Effects of coenzyme Q10 on vascular endothelial function in humans: A meta-analysis of randomized controlled trials. Atherosclerosis.

[8] Fotino A.D., Thompson-Paul A.M., Bazzano L.A. (2013). Effect of coenzyme Q(1)(0) supplementation on heart failure: A meta-analysis. Am. J. Clin. Nutr..

[9] Kosmas C.E., Alkhawam H., El-Hunjul M., Wagman G., Kahn M.R., Grady K.M., Vittorio T.J. (2014). Statin-mediated low-density lipoprotein lowering in chronic congestive heart failure. Am. J. Med. Sci..

[10] Ayer A., Macdonald P., Stocker R. (2015). CoQ(1)(0) Function and Role in Heart Failure and Ischemic Heart Disease. Annu. Rev. Nutr..

[11] Jankowski J., Korzeniowska K., Cieslewicz A., Jablecka A. (2016). Coenzyme Q10—A new player in the treatment of heart failure?. Pharmacol. Rep..

[12] Oleck S., Ventura H.O. (2016). Coenzyme Q10 and Utility in Heart Failure: Just Another Supplement?. Curr. Heart Fail. Rep..

[13] Ba Y.J., Miao Y., Sun Y., Li Z., Cheng G. (2016). Efficacy of coenzyme Q10 as adjuvant therapy in heart failure: A meta-analysis. Chin. J. Evid. Based Med..

[14] Yang Y.K., Wang L.P., Chen L., Yao X.P., Yang K.Q., Gao L.G., Zhou X.L. (2015). Coenzyme Q10 treatment of cardiovascular disorders of ageing including heart failure, hypertension and endothelial dysfunction. Clin. Chim. Acta.

[15] Dludla P.V., Nyambuya T.M., Orlando P., Silvestri S., Mxinwa V., Mokgalaboni K., Nkambule B.B., Louw J., Muller C.J.F., Tiano L. (2020). The impact of coenzyme Q10 on metabolic and cardiovascular disease profiles in diabetic patients: A systematic review and meta-analysis of randomized controlled trials. Endocrinol. Diabetes Metab..

[16] Dludla P.V., Orlando P., Silvestri S., Marcheggiani F., Cirilli I., Nyambuya T.M., Mxinwa V., Mokgalaboni K., Nkambule B.B., Johnson R. (2020). Coenzyme Q10 Supplementation Improves Adipokine Levels and Alleviates Inflammation and Lipid Peroxidation in Conditions of Metabolic Syndrome: A Meta-Analysis of Randomized Controlled Trials. Int. J. Mol. Sci..

[17] Suarez-Rivero J.M., Pastor-Maldonado C.J., de la Mata M., Villanueva-Paz M., Povea-Cabello S., Alvarez-Cordoba M., Villalon-Garcia I., Suarez-Carrillo A., Talaveron-Rey M., Munuera M. (2019). Atherosclerosis and Coenzyme Q10. Int. J. Mol. Sci..

[18] Dragan S., Buleu F., Christodorescu R., Cobzariu F., Iurciuc S., Velimirovici D., Xiao J., Luca C.T. (2019). Benefits of multiple micronutrient supplementation in heart failure: A comprehensive review. Crit. Rev. Food Sci. Nutr..

[19] Dludla P.V., Orlando P., Silvestri S., Mazibuko-Mbeje S.E., Johnson R., Marcheggiani F., Cirilli I., Muller C.J.F., Louw J., Obonye N. (2019). N-Acetyl cysteine ameliorates hyperglycemia-induced cardiomyocyte toxicity by improving mitochondrial energetics and enhancing endogenous Coenzyme Q9/10 levels. Toxicol. Rep..

[20] Dludla P.V., Silvestri S., Orlando P., Mazibuko-Mbeje S.E., Johnson R., Marcheggiani F., Cirilli I., Muller C.J.F., Louw J., Chellan N. (2020). Palmitate-induced toxicity is associated with impaired mitochondrial respiration and accelerated oxidative stress in cultured cardiomyocytes: The critical role of coenzyme Q9/10. Toxicol. In Vitro.

[21] Koziel A., Woyda-Ploszczyca A., Kicinska A., Jarmuszkiewicz W. (2012). The influence of high glucose on the aerobic metabolism of endothelial EA.hy926 cells. Pflugers Arch..

[22] Cirilli I., Orlando P., Marcheggiani F., Dludla P.V., Silvestri S., Damiani E., Tiano L. (2020). The Protective Role of Bioactive Quinones in Stress-induced Senescence Phenotype of Endothelial Cells Exposed to Cigarette Smoke Extract. Antioxidants.

[23] Olivieri F., Lazzarini R., Babini L., Prattichizzo F., Rippo M.R., Tiano L., Di Nuzzo S., Graciotti L., Festa R., Bruge F. (2013). Anti-inflammatory effect of ubiquinol-10 on young and senescent endothelial cells via miR-146a modulation. Free Radic. Biol. Med..

[24] Tsai K.L., Chen L.H., Chiou S.H., Chiou G.Y., Chen Y.C., Chou H.Y., Chen L.K., Chen H.Y., Chiu T.H., Tsai C.S. (2011). Coenzyme Q10 suppresses oxLDL-induced endothelial oxidative injuries by the modulation of LOX-1-mediated ROS generation via the AMPK/PKC/NADPH oxidase signaling pathway. Mol. Nutr. Food Res..

[25] Tsuneki H., Tokai E., Suzuki T., Seki T., Okubo K., Wada T., Okamoto T., Koya S., Kimura I., Sasaoka T. (2013). Protective effects of coenzyme Q10 against angiotensin II-induced oxidative stress in human umbilical vein endothelial cells. Eur. J. Pharmacol..

[26] Alehagen U., Johansson P., Aaseth J., Alexander J., Wagsater D. (2017). Significant changes in circulating microRNA by dietary supplementation of selenium and coenzyme Q10 in healthy elderly males. A subgroup analysis of a prospective randomized double-blind placebo-controlled trial among elderly Swedish citizens. PLoS ONE.

[27] Nemec Svete A., Verk B., Jazbec Križman P., Druzhaeva N., Bohanec A., Petrič D. (2020). Blood variables associated with survival in canine congestive heart failure patients. Bulg. J. Vet. Med..

[28] Svete A.N., Verk B., Seliskar A., Tomsic K., Krizman P.J., Petric A.D. (2017). Plasma coenzyme Q10 concentration, antioxidant status, and serum N-terminal pro-brain natriuretic peptide concentration in dogs with various cardiovascular diseases and the effect of cardiac treatment on measured variables. Am. J. Vet. Res..

[29] Gvozdjakova A., Kucharska J., Kura B., Vancova O., Rausova Z., Sumbalova Z., Ulicna O., Slezak J. (2020). A new insight into the molecular hydrogen effect on coenzyme Q and mitochondrial function of rats. Can. J. Physiol. Pharmacol..

[30] Erol B., Bozlu M., Hanci V., Tokgoz H., Bektas S., Mungan G. (2010). Coenzyme Q10 treatment reduces lipid peroxidation, inducible and endothelial nitric oxide synthases, and germ cell-specific apoptosis in a rat model of testicular ischemia/reperfusion injury. Fertil. Steril..

[31] Nasoohi S., Simani L., Khodagholi F., Nikseresht S., Faizi M., Naderi N. (2019). Coenzyme Q10 supplementation improves acute outcomes of stroke in rats pretreated with atorvastatin. Nutr. Neurosci..

[32] Tillman P., Mann K., Agu R.U., Yeung P.K. (2019). Effect of coenzyme Q10 on ATP metabolism in red blood cells and cardiovascular hemodynamics in an awaken rat model. Curr. Top. Pharmacol..

[33] Shamardl H.A., El-Ashmony S.M., Kamel H.F., Fatani S.H. (2017). Potential Cardiovascular and Renal Protective Effects of Vitamin D and Coenzyme Q10 in l-NAME-Induced Hypertensive Rats. Am. J. Med. Sci..

[34] Kozaeva L.P., Gorodetskaya E.A., Ruuge E.K., Kalenikova E.I., Medvedev O.S. (2017). Beneficial effect of coenzyme Q10 injection on nitric oxide -related dilation of the rat aorta. Eur. J. Pharmacol..

[35] Kulyak O.Y., Gorodetskaya E., Kalenikova E.I., Makarova M.N., Pozharitskaya O.N., Medvedev O.S. (2018). Evaluation of cardioprotective efficacy of innovative dosage form of ubiqinol for intravenous administration. Eksperimental’naya Klin. Farmakol..

[36] Botelho A.F.M., Lempek M.R., Branco S., Nogueira M.M., de Almeida M.E., Costa A.G., Freitas T.G., Rocha M., Moreira M.V.L., Barreto T.O. (2020). Coenzyme Q10 Cardioprotective Effects Against Doxorubicin-Induced Cardiotoxicity in Wistar Rat. Cardiovasc. Toxicol..

[37] Ulla A., Mohamed M.K., Sikder B., Rahman A.T., Sumi F.A., Hossain M., Reza H.M., Rahman G.M.S., Alam M.A. (2017). Coenzyme Q10 prevents oxidative stress and fibrosis in isoprenaline induced cardiac remodeling in aged rats. BMC Pharmacol. Toxicol..

[38] Gairola C.G., Howatt D.A., Daugherty A. (2010). Dietary coenzyme Q10 does not protect against cigarette smoke-augmented atherosclerosis in apoE-deficient mice. Free Radic. Biol. Med..

[39] Xie T., Wang C., Jin Y., Meng Q., Liu Q., Wu J., Sun H. (2020). CoenzymeQ10-Induced Activation of AMPK-YAP-OPA1 Pathway Alleviates Atherosclerosis by Improving Mitochondrial Function, Inhibiting Oxidative Stress and Promoting Energy Metabolism. Front. Pharmacol..

[40] Dudylina A.L., Ivanova M.V., Kalatanova A.V., Kalenikova E.I., Makarov V.G., Makarova M.N., Shumaev K.B., Ruuge E.K. (2019). The generation of superoxide radicals by cardiac mitochondria and the antioxidant effect of the water-soluble form of ubiquinol-10. Cell Biophys..

[41] Gharib A., De Paulis D., Li B., Augeul L., Couture-Lepetit E., Gomez L., Angoulvant D., Ovize M. (2012). Opposite and tissue-specific effects of coenzyme Q2 on mPTP opening and ROS production between heart and liver mitochondria: Role of complex I. J. Mol. Cell Cardiol..

[42] Giorgi C., Agnoletto C., Bononi A., Bonora M., De Marchi E., Marchi S., Missiroli S., Patergnani S., Poletti F., Rimessi A. (2012). Mitochondrial calcium homeostasis as potential target for mitochondrial medicine. Mitochondrion.

[43] Mortensen S.A., Rosenfeldt F., Kumar A., Dolliner P., Filipiak K.J., Pella D., Alehagen U., Steurer G., Littarru G.P., Q-SYMBIO Study Investigators (2014). The effect of coenzyme Q10 on morbidity and mortality in chronic heart failure: Results from Q-SYMBIO: A randomized double-blind trial. JACC Heart Fail..

[44] Mortensen A.L., Rosenfeldt F., Filipiak K.J. (2019). Effect of coenzyme Q10 in Europeans with chronic heart failure: A sub-group analysis of the Q-SYMBIO randomized double-blind trial. Cardiol. J..

[45] Zhao Q., Kebbati A.H., Zhang Y., Tang Y., Okello E., Huang C. (2015). Effect of coenzyme Q10 on the incidence of atrial fibrillation in patients with heart failure. J. Investig. Med..

[46] Alehagen U., Aaseth J., Johansson P. (2015). Reduced Cardiovascular Mortality 10 Years after Supplementation with Selenium and Coenzyme Q10 for Four Years: Follow-Up Results of a Prospective Randomized Double-Blind Placebo-Controlled Trial in Elderly Citizens. PLoS ONE.

[47] Lee B.J., Yen C.H., Hsu H.C., Lin J.Y., Hsia S., Lin P.T. (2012). A significant correlation between the plasma levels of coenzyme Q10 and vitamin B-6 and a reduced risk of coronary artery disease. Nutr. Res..

[48] Lee B.J., Huang Y.C., Chen S.J., Lin P.T. (2012). Coenzyme Q10 supplementation reduces oxidative stress and increases antioxidant enzyme activity in patients with coronary artery disease. Nutrition.

[49] Lee B.J., Tseng Y.F., Yen C.H., Lin P.T. (2013). Effects of coenzyme Q10 supplementation (300 mg/day) on antioxidation and anti-inflammation in coronary artery disease patients during statins therapy: A randomized, placebo-controlled trial. Nutr. J..

[50] Orlando P., Sabbatinelli J., Silvestri S., Marcheggiani F., Cirilli I., Dludla P.V., Molardi A., Nicolini F., Tiano L. (2020). Ubiquinol supplementation in elderly patients undergoing aortic valve replacement: Biochemical and clinical aspects. Aging.

[51] Bagheri Nesami N., Mozaffari-Khosravi H., Najarzadeh A., Salehifar E. (2015). The Effect of Coenzyme Q10 Supplementation on Pro-Inflammatory Factors and Adiponectin in Mildly Hypertensive Patients: A Randomized, Double-Blind, Placebo-Controlled Trial. Int. J. Vitam. Nutr. Res..

[52] Mohseni M., Vafa M., Zarrati M., Shidfar F., Hajimiresmail S.J., Rahimi Forushani A. (2015). Beneficial Effects of Coenzyme Q10 Supplementation on Lipid Profile and Intereukin-6 and Intercellular Adhesion Molecule-1 Reduction, Preliminary Results of a Double-blind Trial in Acute Myocardial Infarction. Int. J. Prev. Med..

[53] Endemann D.H., Schiffrin E.L. (2004). Endothelial dysfunction. J. Am. Soc. Nephrol..

[54] Dai Y.L., Luk T.H., Yiu K.H., Wang M., Yip P.M., Lee S.W., Li S.W., Tam S., Fong B., Lau C.P. (2011). Reversal of mitochondrial dysfunction by coenzyme Q10 supplement improves endothelial function in patients with ischaemic left ventricular systolic dysfunction: A randomized controlled trial. Atherosclerosis.

[55] Kawashima C., Matsuzawa Y., Konishi M., Akiyama E., Suzuki H., Sato R., Nakahashi H., Kikuchi S., Kimura Y., Maejima N. (2020). Ubiquinol Improves Endothelial Function in Patients with Heart Failure with Reduced Ejection Fraction: A Single-Center, Randomized Double-Blind Placebo-Controlled Crossover Pilot Study. Am. J. Cardiovasc/Drugs.

[56] Perez-Sanchez C., Ruiz-Limon P., Aguirre M.A., Bertolaccini M.L., Khamashta M.A., Rodriguez-Ariza A., Segui P., Collantes-Estevez E., Barbarroja N., Khraiwesh H. (2012). Mitochondrial dysfunction in antiphospholipid syndrome: Implications in the pathogenesis of the disease and effects of coenzyme Q(10) treatment. Blood.

[57] Raitakari O.T., McCredie R.J., Witting P., Griffiths K.A., Letters J., Sullivan D., Stocker R., Celermajer D.S. (2000). Coenzyme Q improves LDL resistance to ex vivo oxidation but does not enhance endothelial function in hypercholesterolemic young adults. Free Radic. Biol. Med..

[58] Sabbatinelli J., Orlando P., Galeazzi R., Silvestri S., Cirilli I., Marcheggiani F., Dludla P.V., Giuliani A., Bonfigli A.R., Mazzanti L. (2020). Ubiquinol Ameliorates Endothelial Dysfunction in Subjects with Mild-to-Moderate Dyslipidemia: A Randomized Clinical Trial. Nutrients.

[59] Langsjoen P.H., Langsjoen A.M. (2014). Comparison study of plasma coenzyme Q10 levels in healthy subjects supplemented with ubiquinol versus ubiquinone. Clin. Pharmacol. Drug Dev..

[60] Tournaye H. (2006). Evidence-based management of male subfertility. Curr. Opin. Obstet. Gynecol..

[61] Wang Q., Sun Q.Y. (2007). Evaluation of oocyte quality: Morphological, cellular and molecular predictors. Reprod. Fertil. Dev..

[62] Bykova M., Athayde K., Sharma R., Jha R., Sabanegh E., Agarwal A. (2007). Defining the reference value of seminal reactive oxygen species in a population of infertile men and normal healthy volunteers. Fertil. Steril..

[63] Miao Y.L., Kikuchi K., Sun Q.Y., Schatten H. (2009). Oocyte aging: Cellular and molecular changes, developmental potential and reversal possibility. Hum. Reprod. Update.

[64] Tremellen K. (2008). Oxidative stress and male infertility—A clinical perspective. Hum. Reprod. Update.

[65] Alahmar A.T., Calogero A.E., Sengupta P., Dutta S. (2021). Coenzyme Q10 Improves Sperm Parameters, Oxidative Stress Markers and Sperm DNA Fragmentation in Infertile Patients with Idiopathic Oligoasthenozoospermia. World J. Men’s Health.

[66] Nadjarzadeh A., Shidfar F., Amirjannati N., Vafa M.R., Motevalian S.A., Gohari M.R., Nazeri Kakhki S.A., Akhondi M.M., Sadeghi M.R. (2014). Effect of Coenzyme Q10 supplementation on antioxidant enzymes activity and oxidative stress of seminal plasma: A double-blind randomised clinical trial. Andrologia.

[67] Cakiroglu B., Eyyupoglu S.E., Gozukucuk R., Uyanik B.S. (2014). Ubiquinol effect on sperm parameters in subfertile men who have astheno-teratozoospermia with normal sperm concentration. Nephrourol. Mon..

[68] Safarinejad M.R., Safarinejad S., Shafiei N., Safarinejad S. (2012). Effects of the reduced form of coenzyme Q10 (ubiquinol) on semen parameters in men with idiopathic infertility: A double-blind, placebo controlled, randomized study. J. Urol..

[69] Tirabassi G., Vignini A., Tiano L., Buldreghini E., Bruge F., Silvestri S., Orlando P., D’Aniello A., Mazzanti L., Lenzi A. (2015). Protective effects of coenzyme Q10 and aspartic acid on oxidative stress and DNA damage in subjects affected by idiopathic asthenozoospermia. Endocrine.

[70] Safarinejad M.R. (2012). The effect of coenzyme Q(1)(0) supplementation on partner pregnancy rate in infertile men with idiopathic oligoasthenoteratozoospermia: An open-label prospective study. Int. Urol. Nephrol..

[71] Gvozdjakova A., Kucharska J., Dubravicky J., Mojto V., Singh R.B. (2015). Coenzyme Q(1)(0), alpha-tocopherol, and oxidative stress could be important metabolic biomarkers of male infertility. Dis. Mark..

[72] Kobori Y., Ota S., Sato R., Yagi H., Soh S., Arai G., Okada H. (2014). Antioxidant cosupplementation therapy with vitamin C, vitamin E, and coenzyme Q10 in patients with oligoasthenozoospermia. Arch. Ital. Urol. Androl..

[73] Giannubilo S.R., Orlando P., Silvestri S., Cirilli I., Marcheggiani F., Ciavattini A., Tiano L. (2018). CoQ10 Supplementation in Patients Undergoing IVF-ET: The Relationship with Follicular Fluid Content and Oocyte Maturity. Antioxidants.

[74] Gat I., Blanco Mejia S., Balakier H., Librach C.L., Claessens A., Ryan E.A. (2016). The use of coenzyme Q10 and DHEA during IUI and IVF cycles in patients with decreased ovarian reserve. Gynecol. Endocrinol..

[75] Xu Y., Nisenblat V., Lu C., Li R., Qiao J., Zhen X., Wang S. (2018). Pretreatment with coenzyme Q10 improves ovarian response and embryo quality in low-prognosis young women with decreased ovarian reserve: A randomized controlled trial. Reprod. Biol. Endocrinol..

[76] Bentov Y., Hannam T., Jurisicova A., Esfandiari N., Casper R.F. (2014). Coenzyme Q10 Supplementation and Oocyte Aneuploidy in Women Undergoing IVF-ICSI Treatment. Clin. Med. Insights Reprod. Health.

[77] Said R.S., Mohamed H.A., Kamal M.M. (2019). Coenzyme Q10 mitigates ionizing radiation-induced testicular damage in rats through inhibition of oxidative stress and mitochondria-mediated apoptotic cell death. Toxicol. Appl. Pharmacol..

[78] Delkhosh A., Delashoub M., Tehrani A.A., Bahrami A.M., Niazi V., Shoorei H., Banimohammad M., Kalarestaghi H., Shokoohi M., Agabalazadeh A. (2019). Upregulation of FSHR and PCNA by administration of coenzyme Q10 on cyclophosphamide-induced premature ovarian failure in a mouse model. J. Biochem. Mol. Toxicol..

[79] Zhang M., ShiYang X., Zhang Y., Miao Y., Chen Y., Cui Z., Xiong B. (2019). Coenzyme Q10 ameliorates the quality of postovulatory aged oocytes by suppressing DNA damage and apoptosis. Free Radic. Biol. Med..

[80] Niu Y.J., Zhou W., Nie Z.W., Zhou D., Xu Y.N., Ock S.A., Yan C.G., Cui X.S. (2020). Ubiquinol-10 delays postovulatory oocyte aging by improving mitochondrial renewal in pigs. Aging.

[81] Ben-Meir A., Burstein E., Borrego-Alvarez A., Chong J., Wong E., Yavorska T., Naranian T., Chi M., Wang Y., Bentov Y. (2015). Coenzyme Q10 restores oocyte mitochondrial function and fertility during reproductive aging. Aging Cell.

[82] Radak Z., Zhao Z., Koltai E., Ohno H., Atalay M. (2013). Oxygen consumption and usage during physical exercise: The balance between oxidative stress and ROS-dependent adaptive signaling. Antioxid. Redox Signal..

[83] Guescini M., Tiano L., Genova M.L., Polidori E., Silvestri S., Orlando P., Fimognari C., Calcabrini C., Stocchi V., Sestili P. (2017). The Combination of Physical Exercise with Muscle-Directed Antioxidants to Counteract Sarcopenia: A Biomedical Rationale for Pleiotropic Treatment with Creatine and Coenzyme Q10. Oxid. Med. Cell Longev..

[84] Orlando P., Silvestri S., Galeazzi R., Antonicelli R., Marcheggiani F., Cirilli I., Bacchetti T., Tiano L. (2018). Effect of ubiquinol supplementation on biochemical and oxidative stress indexes after intense exercise in young athletes. Redox Rep..

[85] Fischer A., Onur S., Niklowitz P., Menke T., Laudes M., Rimbach G., Doring F. (2016). Coenzyme Q10 Status as a Determinant of Muscular Strength in Two Independent Cohorts. PLoS ONE.

[86] Cruz-Jentoft A.J., Bahat G., Bauer J., Boirie Y., Bruyere O., Cederholm T., Cooper C., Landi F., Rolland Y., Sayer A.A. (2019). Sarcopenia: Revised European consensus on definition and diagnosis. Age Ageing.

[87] Andreani C., Bartolacci C., Guescini M., Battistelli M., Stocchi V., Orlando F., Provinciali M., Amici A., Marchini C., Tiano L. (2018). Combination of Coenzyme Q10 Intake and Moderate Physical Activity Counteracts Mitochondrial Dysfunctions in a SAMP8 Mouse Model. Oxid. Med. Cell Longev..

[88] Christiansen L.B., Dohlmann T.L., Ludvigsen T.P., Parfieniuk E., Ciborowski M., Szczerbinski L., Kretowski A., Desler C., Tiano L., Orlando P. (2021). Atorvastatin impairs liver mitochondrial function in obese Gottingen Minipigs but heart and skeletal muscle are not affected. Sci. Rep..

[89] La Guardia P.G., Alberici L.C., Ravagnani F.G., Catharino R.R., Vercesi A.E. (2013). Protection of rat skeletal muscle fibers by either L-carnitine or coenzyme Q10 against statins toxicity mediated by mitochondrial reactive oxygen generation. Front. Physiol..

[90] Wang L.W., Jabbour A., Hayward C.S., Furlong T.J., Girgis L., Macdonald P.S., Keogh A.M. (2015). Potential role of coenzyme Q10 in facilitating recovery from statin-induced rhabdomyolysis. Intern. Med. J..

[91] Derosa G., D’Angelo A., Maffioli P. (2019). Coenzyme q10 liquid supplementation in dyslipidemic subjects with statin-related clinical symptoms: A double-blind, randomized, placebo-controlled study. Drug Des. Devel. Ther..

[92] Muraki A., Miyashita K., Mitsuishi M., Tamaki M., Tanaka K., Itoh H. (2012). Coenzyme Q10 reverses mitochondrial dysfunction in atorvastatin-treated mice and increases exercise endurance. J. Appl. Physiol. (1985).

[93] Piercy K.L., Troiano R.P., Ballard R.M., Carlson S.A., Fulton J.E., Galuska D.A., George S.M., Olson R.D. (2018). The Physical Activity Guidelines for Americans. JAMA.

[94] Simon H.B. (2015). Exercise and Health: Dose and Response, Considering Both Ends of the Curve. Am. J. Med..

[95] Diaz-Castro J., Moreno-Fernandez J., Chirosa I., Chirosa L.J., Guisado R., Ochoa J.J. (2020). Beneficial Effect of Ubiquinol on Hematological and Inflammatory Signaling during Exercise. Nutrients.

[96] Suzuki K., Yamada M., Kurakake S., Okamura N., Yamaya K., Liu Q., Kudoh S., Kowatari K., Nakaji S., Sugawara K. (2000). Circulating cytokines and hormones with immunosuppressive but neutrophil-priming potentials rise after endurance exercise in humans. Eur. J. Appl. Physiol..

[97] Chen H.C., Huang C.C., Lin T.J., Hsu M.C., Hsu Y.J. (2019). Ubiquinol Supplementation Alters Exercise Induced Fatigue by Increasing Lipid Utilization in Mice. Nutrients.

[98] Sarmiento A., Diaz-Castro J., Pulido-Moran M., Moreno-Fernandez J., Kajarabille N., Chirosa I., Guisado I.M., Javier Chirosa L., Guisado R., Ochoa J.J. (2016). Short-term ubiquinol supplementation reduces oxidative stress associated with strenuous exercise in healthy adults: A randomized trial. Biofactors.

[99] Bloomer R.J., Canale R.E., McCarthy C.G., Farney T.M. (2012). Impact of oral ubiquinol on blood oxidative stress and exercise performance. Oxid. Med. Cell Longev..

[100] Gul I., Gokbel H., Belviranli M., Okudan N., Buyukbas S., Basarali K. (2011). Oxidative stress and antioxidant defense in plasma after repeated bouts of supramaximal exercise: The effect of coenzyme Q10. J. Sports Med. Phys. Fitness.

[101] Emmanuele V., Lopez L.C., Berardo A., Naini A., Tadesse S., Wen B., D’Agostino E., Solomon M., DiMauro S., Quinzii C. (2012). Heterogeneity of coenzyme Q10 deficiency: Patient study and literature review. Arch. Neurol..

[102] Yubero D., Montero R., Martin M.A., Montoya J., Ribes A., Grazina M., Trevisson E., Rodriguez-Aguilera J.C., Hargreaves I.P., Salviati L. (2016). Secondary coenzyme Q10 deficiencies in oxidative phosphorylation (OXPHOS) and non-OXPHOS disorders. Mitochondrion.

[103] Berardo A., Quinzii C.M. (2020). Redefining infantile-onset multisystem phenotypes of coenzyme Q10-deficiency in the next-generation sequencing era. J. Transl. Genet. Genom..

[104] Salviati L., Trevisson E., Doimo M., Navas P., Adam M.P., Ardinger H.H., Pagon R.A., Wallace S.E., Bean L.J.H., Mirzaa G., Amemiya A. (2017). Primary Coenzyme Q10 Deficiency. GeneReviews((R)).

[105] Montero R., Yubero D., Salgado M.C., Gonzalez M.J., Campistol J., O’Callaghan M.D.M., Pineda M., Delgadillo V., Maynou J., Fernandez G. (2019). Plasma coenzyme Q10 status is impaired in selected genetic conditions. Sci. Rep..

[106] Neergheen V., Hargreaves I.P., Grigoryeva S. (2018). Secondary Coenzyme Q10 Deficiency: Causes and Consequence.

[107] Yubero D., Montero R., Artuch R., Land J.M., Heales S.J., Hargreaves I.P. (2014). Biochemical diagnosis of coenzyme q10 deficiency. Mol. Syndromol..

[108] Wainwright L., Hargreaves I.P., Georgian A.R., Turner C., Dalton R.N., Abbott N.J., Heales S.J.R., Preston J.E. (2020). CoQ10 Deficient Endothelial Cell Culture Model for the Investigation of CoQ10 Blood-Brain Barrier Transport. J. Clin. Med..

[109] Mancuso M., Orsucci D., Volpi L., Calsolaro V., Siciliano G. (2010). Coenzyme Q10 in neuromuscular and neurodegenerative disorders. Curr. Drug Targets.

[110] Salama M., Yuan T.F., Machado S., Murillo-Rodriguez E., Vega J.A., Menendez-Gonzalez M., Nardi A.E., Arias-Carrion O. (2013). Co-enzyme Q10 to treat neurological disorders: Basic mechanisms, clinical outcomes, and future research direction. CNS Neurol. Disord. Drug Targets.

[111] Duran-Prado M., Frontinan J., Santiago-Mora R., Peinado J.R., Parrado-Fernandez C., Gomez-Almagro M.V., Moreno M., Lopez-Dominguez J.A., Villalba J.M., Alcain F.J. (2014). Coenzyme Q10 protects human endothelial cells from beta-amyloid uptake and oxidative stress-induced injury. PLoS ONE.

[112] Komaki H., Faraji N., Komaki A., Shahidi S., Etaee F., Raoufi S., Mirzaei F. (2019). Investigation of protective effects of coenzyme Q10 on impaired synaptic plasticity in a male rat model of Alzheimer’s disease. Brain Res. Bull..

[113] Zaki M.E., El-Bassyouni H.T., Tosson A.M., Youness E., Hussein J. (2017). Coenzyme Q10 and pro-inflammatory markers in children with Down syndrome: Clinical and biochemical aspects. J. Pediatr. Rio J..

[114] Tiano L., Carnevali P., Padella L., Santoro L., Principi F., Bruge F., Carle F., Gesuita R., Gabrielli O., Littarru G.P. (2011). Effect of Coenzyme Q10 in mitigating oxidative DNA damage in Down syndrome patients, a double blind randomized controlled trial. Neurobiol. Aging.

[115] Larsen E.L., Padella L., Bergholdt H.K.M., Henriksen T., Santoro L., Gabrielli O., Poulsen H.E., Littarru G.P., Orlando P., Tiano L. (2018). The effect of long-term treatment with coenzyme Q10 on nucleic acid modifications by oxidation in children with Down syndrome. Neurobiol. Aging.

[116] Miles M.V., Patterson B.J., Chalfonte-Evans M.L., Horn P.S., Hickey F.J., Schapiro M.B., Steele P.E., Tang P.H., Hotze S.L. (2007). Coenzyme Q10 (ubiquinol-10) supplementation improves oxidative imbalance in children with trisomy 21. Pediatr. Neurol..

[117] Chang K.H., Chen C.M. (2020). The Role of Oxidative Stress in Parkinson’s Disease. Antioxidants.

[118] Park H.W., Park C.G., Park M., Lee S.H., Park H.R., Lim J., Paek S.H., Choy Y.B. (2020). Intrastriatal administration of coenzyme Q10 enhances neuroprotection in a Parkinson’s disease rat model. Sci. Rep..

[119] Sanoobar M., Eghtesadi S., Azimi A., Khalili M., Jazayeri S., Reza Gohari M. (2013). Coenzyme Q10 supplementation reduces oxidative stress and increases antioxidant enzyme activity in patients with relapsing-remitting multiple sclerosis. Int. J. Neurosci..

[120] Garcia-Corzo L., Luna-Sanchez M., Doerrier C., Ortiz F., Escames G., Acuna-Castroviejo D., Lopez L.C. (2014). Ubiquinol-10 ameliorates mitochondrial encephalopathy associated with CoQ deficiency. Biochim. Biophys. Acta.

[121] Gupta B.K., Kumar S., Kaur H., Ali J., Baboota S. (2018). Attenuation of Oxidative Damage by Coenzyme Q 10 Loaded Nanoemulsion Through Oral Route for the Management of Parkinson’s Disease. Rejuvenation Res..

[122] Sadeghiyan Galeshkalami N., Abdollahi M., Najafi R., Baeeri M., Jamshidzade A., Falak R., Davoodzadeh Gholami M., Hassanzadeh G., Mokhtari T., Hassani S. (2019). Alpha-lipoic acid and coenzyme Q10 combination ameliorates experimental diabetic neuropathy by modulating oxidative stress and apoptosis. Life Sci..

[123] Shi T.J., Zhang M.D., Zeberg H., Nilsson J., Grunler J., Liu S.X., Xiang Q., Persson J., Fried K.J., Catrina S.B. (2013). Coenzyme Q10 prevents peripheral neuropathy and attenuates neuron loss in the db-/db- mouse, a type 2 diabetes model. Proc. Natl. Acad. Sci. USA.

[124] Zhang Y.P., Eber A., Yuan Y., Yang Z., Rodriguez Y., Levitt R.C., Takacs P., Candiotti K.A. (2013). Prophylactic and antinociceptive effects of coenzyme Q10 on diabetic neuropathic pain in a mouse model of type 1 diabetes. Anesthesiology.

[125] Ahmadvand H., Tavafi M., Khosrowbeygi A. (2012). Amelioration of altered antioxidant enzymes activity and glomerulosclerosis by coenzyme Q10 in alloxan-induced diabetic rats. J. Diabetes Complicat..

[126] Tsai H.Y., Lin C.P., Huang P.H., Li S.Y., Chen J.S., Lin F.Y., Chen J.W., Lin S.J. (2016). Coenzyme Q10 Attenuates High Glucose-Induced Endothelial Progenitor Cell Dysfunction through AMP-Activated Protein Kinase Pathways. J. Diabetes Res..

[127] Sun J., Zhu H., Wang X., Gao Q., Li Z., Huang H. (2019). CoQ10 ameliorates mitochondrial dysfunction in diabetic nephropathy through mitophagy. Endocrinology.

[128] Tarry-Adkins J.L., Fernandez-Twinn D.S., Madsen R., Chen J.H., Carpenter A., Hargreaves I.P., McConnell J.M., Ozanne S.E. (2015). Coenzyme Q10 Prevents Insulin Signaling Dysregulation and Inflammation Prior to Development of Insulin Resistance in Male Offspring of a Rat Model of Poor Maternal Nutrition and Accelerated Postnatal Growth. Endocrinology.

[129] Ustuner M.A., Kaman D., Colakoglu N. (2017). Effects of benfotiamine and coenzyme Q10 on kidney damage induced gentamicin. Tissue Cell.

[130] Carrasco J., Anglada F.J., Campos J.P., Muntane J., Requena M.J., Padillo J. (2014). The protective role of coenzyme Q10 in renal injury associated with extracorporeal shockwave lithotripsy: A randomised, placebo-controlled clinical trial. BJU Int..

[131] Gao J.J., Xu Y.X., Jia H.P., Zhang L., Cao X.Y., Zuo X.W., Cai G.Y., Chen X.M. (2021). Associations of coenzyme Q10 with endothelial function in hemodialysis patients. Nephrology.

[132] Farsi F., Mohammadshahi M., Alavinejad P., Rezazadeh A., Zarei M., Engali K.A. (2016). Functions of coenzyme Q10 supplementation on liver enzymes, markers of systemic inflammation, and adipokines in patients affected by nonalcoholic fatty liver disease: A double-blind, placebo-controlled, randomized clinical trial. J. Am. Coll. Nutr..

[133] Mohamed HA S.R. (2021). Coenzyme Q10 attenuates inflammation and fibrosis implicated in radiation enteropathy through suppression of NF-kB/TGF-β/MMP-9 pathways. Int. Immunopharmacol..

[134] El-Sheikh A.A., Morsy M.A., Mahmoud M.M., Rifaai R.A. (2014). Protective mechanisms of coenzyme-Q10 may involve up-regulation of testicular P-glycoprotein in doxorubicin-induced toxicity. Environ. Toxicol. Pharmacol..

[135] Noh Y.H., Kim K.Y., Shim M.S., Choi S.H., Choi S., Ellisman M.H., Weinreb R.N., Perkins G.A., Ju W.K. (2013). Inhibition of oxidative stress by coenzyme Q10 increases mitochondrial mass and improves bioenergetic function in optic nerve head astrocytes. Cell Death Dis..

[136] Lee D., Shim M.S., Kim K.Y., Noh Y.H., Kim H., Kim S.Y., Weinreb R.N., Ju W.K. (2014). Coenzyme Q10 inhibits glutamate excitotoxicity and oxidative stress-mediated mitochondrial alteration in a mouse model of glaucoma. Investig. Ophthalmol. Vis. Sci..

[137] Parisi V., Centofanti M., Gandolfi S., Marangoni D., Rossetti L., Tanga L., Tardini M., Traina S., Ungaro N., Vetrugno M. (2014). Effects of coenzyme Q10 in conjunction with vitamin E on retinal-evoked and cortical-evoked responses in patients with open-angle glaucoma. J. Glaucoma.

[138] Arranz-Romera A., Davis B., Bravo-Osuna I., Esteban-Pérez S., Molina-Martínez I.T., Shamsher E., Ravindran N., Guo L., Cordeiro M.F., Herrero-Vanrell R. (2019). Simultaneous co-delivery of neuroprotective drugs from multi-loaded PLGA microspheres for the treatment of glaucoma. J. Control. Release.

[139] Mencucci R., Favuzza E., Boccalini C., Lapucci A., Felici R., Resta F., Chiarugi A., Cavone L. (2014). CoQ10-containing eye drops prevent UVB-induced cornea cell damage and increase cornea wound healing by preserving mitochondrial function. Investig. Ophthalmol. Vis. Sci..

[140] Lee D., Kim K.Y., Shim M.S., Kim S.Y., Ellisman M.H., Weinreb R.N., Ju W.K. (2014). Coenzyme Q10 ameliorates oxidative stress and prevents mitochondrial alteration in ischemic retinal injury. Apoptosis.

[141] Fernández-Vega B., Nicieza J., Álvarez-Barrios A., Álvarez L., García M., Fernández-Vega C., Vega J.A., González-Iglesias H. (2020). The Use of Vitamins and Coenzyme Q10 for the Treatment of Vascular Occlusion Diseases Affecting the Retina. Nutrients.

[142] Cevenini E., Invidia L., Lescai F., Salvioli S., Tieri P., Castellani G., Franceschi C. (2008). Human models of aging and longevity. Expert Opin. Biol. Ther..

[143] Pardeike J., Schwabe K., Muller R.H. (2010). Influence of nanostructured lipid carriers (NLC) on the physical properties of the Cutanova Nanorepair Q10 cream and the in vivo skin hydration effect. Int. J. Pharm..

[144] Shindo Y., Witt E., Han D., Epstein W., Packer L. (1994). Enzymic and non-enzymic antioxidants in epidermis and dermis of human skin. J. Investg. Dermatol..

[145] Kalen A., Appelkvist E.L., Dallner G. (1989). Age-related changes in the lipid compositions of rat and human tissues. Lipids.

[146] Passi S., De Pita O., Puddu P., Littarru G.P. (2002). Lipophilic antioxidants in human sebum and aging. Free Radic. Res..

[147] Ely J.T., Krone C.A. (2000). A Brief Update on Ubiquinone Coenzyme Q10. J. Orthomol. Med..

[148] Marcheggiani F., Cirilli I., Orlando P., Silvestri S., Vogelsang A., Knott A., Blatt T., Weise J.M., Tiano L. (2019). Modulation of Coenzyme Q10 content and oxidative status in human dermal fibroblasts using HMG-CoA reductase inhibitor over a broad range of concentrations. From mitohormesis to mitochondrial dysfunction and accelerated aging. Aging.

[149] Marcheggiani F., Kordes S., Cirilli I., Orlando P., Silvestri S., Vogelsang A., Moller N., Blatt T., Weise J.M., Damiani E. (2021). Anti-ageing effects of ubiquinone and ubiquinol in a senescence model of human dermal fibroblasts. Free Radic. Biol. Med..

[150] Zmitek K., Zmitek J., Rogl Butina M., Pogacnik T. (2020). Effects of a Combination of Water-Soluble CoenzymeQ10 and Collagen on Skin Parameters and Condition:Results of a Randomised, Placebo-Controlled, Double-Blind Study. Nutrients.

[151] Zmitek K., Pogacnik T., Mervic L., Zmitek J., Pravst I. (2017). The effect of dietary intake of coenzyme Q10 on skin parameters and condition: Results of a randomised, placebo-controlled, double-blind study. Biofactors.

[152] Schniertshauer D., Gebhard D., Bergemann J. (2018). Age-Dependent Loss of Mitochondrial Function in Epithelial Tissue Can Be Reversed by Coenzyme Q10. J. Aging Res..

[153] Knott A., Achterberg V., Smuda C., Mielke H., Sperling G., Dunckelmann K., Vogelsang A., Kruger A., Schwengler H., Behtash M. (2015). Topical treatment with coenzyme Q10-containing formulas improves skin’s Q10 level and provides antioxidative effects. Biofactors.

[154] Yue Y., Zhou H., Liu G., Li Y., Yan Z., Duan M. (2010). The advantages of a novel CoQ10 delivery system in skin photo-protection. Int. J. Pharm..

[155] Bruge F., Damiani E., Puglia C., Offerta A., Armeni T., Littarru G.P., Tiano L. (2013). Nanostructured lipid carriers loaded with CoQ10: Effect on human dermal fibroblasts under normal and UVA-mediated oxidative conditions. Int. J. Pharm..

[156] El-Leithy E.S., Makky A.M., Khattab A.M., Hussein D.G. (2018). Optimization of nutraceutical coenzyme Q10 nanoemulsion with improved skin permeability and anti-wrinkle efficiency. Drug Dev. Ind. Pharm..

[157] Zhang M., Dang L., Guo F., Wang X., Zhao W., Zhao R. (2012). Coenzyme Q10 enhances dermal elastin expression, inhibits IL-1α production and melanin synthesis in vitro. Int. J. Cosmet. Sci..

[158] Kurban S., Mehmetoglu I. (2010). Effects of acetylsalicylic acid on serum paraoxonase activity, Ox-LDL, coenzyme Q10 and other oxidative stress markers in healthy volunteers. Clin. Biochem..

[159] Fumagalli S., Fattirolli F., Guarducci L., Cellai T., Baldasseroni S., Tarantini F., Di Bari M., Masotti G., Marchionni N. (2011). Coenzyme Q_10_ terclatrate and creatine in chronic heart failure: A randomized, placebo-controlled, double-blind study. Clin. Cardiol..

[160] Mikhin V.P., Kharchenko A.V., Rosliakova E.A., Cherniatina M.A. (2011). Application of coenzyme Q(10) in combination therapy of arterial hypertension. Kardiologiia.

[161] Toyama K., Sugiyama S., Oka H., Iwasaki Y., Tomoko H., Shinji Tayama H., Jinnouchi H., Matsui K., Ogawa H. (2011). Rosuvastatin combined with regular exercise preserves coenzyme Q10 levels associated with a significant increase in high-density lipoprotein cholesterol in patients with coronary artery disease. Atherosclerosis.

[162] Young J.M., Molyneux S.L., Reinheimer A.M., Florkowski C.M., Frampton C.M., Scott R.S., George P.M. (2011). Relationship between plasma coenzyme Q_10_, asymmetric dimethylarginine and arterial stiffness in patients with phenotypic or genotypic familial hypercholesterolemia on long-term statin therapy. Atherosclerosis.

[163] Brugè F., Bacchetti T., Principi F., Scarpa E.S., Littarru G.P., Tiano L. (2012). Olive oil supplemented with Coenzyme Q(10): Effect on plasma and lipoprotein oxidative status. Biofactors.

[164] Larijani N.V., Ahmadi N., Zeb I., Khan F., Flores F., Budoff M. (2013). Beneficial effects of aged garlic extract and coenzyme Q10 on vascular elasticity and endothelial function: The FAITH randomized clinical trial. Nutrition.

[165] Sharp J., Farha S., Park M.M., Comhair S.A., Lundgrin E.L., Tang W.H.W., Bongard R.D., Merker M.P., Erzurum S.C. (2014). Coenzyme Q supplementation in pulmonary arterial hypertension. Redox Biol..

[166] Pérez-Sánchez C., Aguirre M.A., Ruiz-Limón P., Ábalos-Aguilera M.C., Jiménez-Gómez Y., Arias-de la Rosa I., Rodriguez-Ariza A., Fernández-Del Río L., González-Reyes J.A., Segui P. (2017). Ubiquinol Effects on Antiphospholipid Syndrome Prothrombotic Profile: A Randomized, Placebo-Controlled Trial. Arterioscler. Thromb. Vasc. Biol..

[167] Shikh E., Zozina V., Kondratenko S., Melnikov E., Kukes V. (2020). The particulars of certain drugs’ effect on the endogenous coenzyme Q_10_ plasma level in patients with cardiovascular diseases. Drug Metab. Pers. Ther..

